# Synergistic and Antagonistic Effects of Thermal Shock, Air Exposure, and Fishing Capture on the Physiological Stress of *Squilla mantis* (Stomatopoda)

**DOI:** 10.1371/journal.pone.0105060

**Published:** 2014-08-18

**Authors:** Saša Raicevich, Fabrizio Minute, Maria Grazia Finoia, Francesca Caranfa, Paolo Di Muro, Lucia Scapolan, Mariano Beltramini

**Affiliations:** 1 ISPRA - National Institute for Environmental Protection and Research, Chioggia, Italy; 2 Department of Biology, University of Padova, Padova, Italy; 3 ISPRA - National Institute for Environmental Protection and Research, Roma, Italy; University of Waikato (National Institute of Water and Atmospheric Research), New Zealand

## Abstract

This study is aimed at assessing the effects of multiple stressors (thermal shock, fishing capture, and exposure to air) on the benthic stomatopod *Squilla mantis*, a burrowing crustacean quite widespread in the Mediterranean Sea. Laboratory analyses were carried out to explore the physiological impairment onset over time, based on emersion and thermal shocks, on farmed individuals. Parallel field-based studies were carried out to also investigate the role of fishing (i.e., otter trawling) in inducing physiological imbalance in different seasonal conditions. The dynamics of physiological recovery from physiological disruption were also studied. Physiological stress was assessed by analysing hemolymph metabolites (L-Lactate, D-glucose, ammonia, and H^+^), as well as glycogen concentration in muscle tissues. The experiments were carried out according to a factorial scheme considering the three factors (thermal shock, fishing capture, and exposure to air) at two fixed levels in order to explore possible synergistic, additive, or antagonistic effects among factors. Additive effects on physiological parameters were mainly detected when the three factors interacted together while synergistic effects were found as effect of the combination of two factors. This finding highlights that the physiological adaptive and maladaptive processes induced by the stressors result in a dynamic response that may encounter physiological limits when high stress levels are sustained. Thus, a further increase in the physiological parameters due to synergies cannot be reached. Moreover, when critical limits are encountered, mortality occurs and physiological parameters reflect the response of the last survivors. In the light of our mortality studies, thermal shock and exposure to air have the main effect on the survival of *S. mantis* only on trawled individuals, while lab-farmed individuals did not show any mortality during exposure to air until after 2 hours.

## Introduction

In recent years, the study of cumulative effects of multiple stressors on marine species, communities, and ecosystems has attracted much attention from scientists and managers [1]–[5]. While most of the studies carried out until the late 1990s were focused on the assessment of the effects from one or two factors, recent work has shown that the ecological consequences of multiple stressors cannot be always easily predicted (i.e., ecological surprise; [2]) since they may result in synergistic or antagonistic effects. Indeed, two stressors may interact in a synergistic way when their cumulative effects are higher than the sum of the effects of single stressor. Conversely, when the overall effects of multiple stressors are significantly lower than the sum of the effects of the single stressors, antagonism occurs. This complexity is further increased when three or more stressors are taken into account, since the frequency of non-linear responses in the investigated ecological variables sharply increases [1], [5].

From the management perspective, there is a need for increasing scientific knowledge about this topic, because marine species and ecosystems worldwide are subjected to multiple stressors [3]. Thus, it is important to understand what drivers may have the most important impact and which act synergistically in order to select the “optimal” combination of drivers that allow “sustainable” disturbance while still permitting human activities to be carried out. A similar approach is required, for instance, in the implementation of the Marine Strategy Framework Directive, a European legislation that aims to reach the so-called “Good Environmental Status” in European marine waters by 2020 [6]. However, while the scientific knowledge of marine biodiversity has increased in the past decades, a pressing need for a more complete understanding of the cumulative impact of human-induced drivers is advocated by the scientific community [7]. At the same time, the continuously changing environmental conditions of our world's oceans (i.e., global warming, acidification) are likely to induce changes in the distribution and abundance of species. Understanding these changes requires an integrative approach to progress from single-factor analysis to multiple-stressor effect studies [5].

Due to the large variety of laboratory studies and experimental settings, only meta-analytic approaches have been able to shed light on the inherent complexity of multiple stressors interactions at the species level. These studies were mainly based on the analyses of the outcomes of laboratory experiments, since in controlled conditions it is possible to better isolate and modulate the stressors. [1]–[2]. Moreover, general conclusions can be drawn only when studies dealing with multiple drivers are carried out according to a factorial experimental design in order to test the role of interactions.

In the context of fishery ecology, several field and laboratory studies have addressed the role of trawling and handling on the stress and mortality of commercial and non-commercial species [8]–[11]. These studies showed that trawling and handling (mechanical stress), thermal shock and barotrauma (that occurs to specimens displaced from the sea bottom to the deck of fishing vessels), as well as exposure to air (during sorting and handling), may induce adaptive and maladaptive behavioral and physiological responses to stress [11] that can result in high mortality rates. However, most of the research carried out so far lacked a factorial approach and, thus, its capability to understand whether or not these factors acts synergistically and which stressor prevails in inducing physiological imbalance and mortality was reduced. In this context, the combination of laboratory investigations and field experiments under realistic fishing conditions proved to be better suited to understand both the physiological process and prediction of mortality in discarded bycaught species [12].

In order to investigate the cumulative impact and relative roles of thermal shock (i.e., seasonal conditions), exposure to air, and trawling on caught and discarded species, we carried out laboratory and field studies on the benthic stomatopod *Squilla mantis*. This burrowing crustacean is found in high densities in the Mediterranean Sea in areas with suitable burrowing substrates, such as fine sand and sandy mud [13]–[14] and at a depth of 10–200 m. *S. mantis* is the only stomatopod fished for commercial purposes in the Mediterranean Sea and it is caught as a target species or bycatch in demersal multi-target fisheries; it is discarded in some areas due to its negligible local economic value [15]–[16]. A recent analysis of the stress induced by fishing on such species highlighted that *S. mantis* can suffer physiological alteration due to fishery capture, thus confirming that this species can be used as guide species to investigate the presence of synergistic effects among multiple stressors, i.e., thermal shock, air exposure, and fishing capture, in marine crustaceans [17].

For the purposes of our study, two experimental paradigms were applied. Laboratory analyses were carried out to explore the physiological impairment onset over time, as a result of emersion and thermal shocks, on control individuals. Parallel field-based studies were carried out to investigate the role of fishing (i.e., otter trawling) on inducing physiological imbalance in different seasonal conditions. The dynamics of physiological stress onset during post-trawling air emersion and the recovery from physiological disruption were also studied. In all experimental conditions, physiological stress was assessed by analysing hemolymph metabolites (L-lactate, D-glucose, and ammonia) and pH, as well as glycogen concentration in muscle tissues. The experiments were carried out according to a factorial scheme considering three factors (thermal shock, air exposure, and trawling) at two fixed levels. Thus, the data could be also analysed in order to explore the presence of synergistic, additive, or antagonistic effects of such factors on each physiological stress indicator. Multivariate analysis was also applied in order to investigate the overall displacement of stressed individuals from homeostasis according to different experimental conditions. Parallel survival tests in control and trawled and emersed specimens under different seasons also enabled the delineation of critical limits of stressors [18] that point to maladaptive responses and the onset of physiological disruptions in *S. mantis*.

## Materials and Methods

### Sampling and environmental characteristics

*Squilla mantis* individuals were collected during commercial tows in the Northern Adriatic Sea approximately 6–10 km off the Italian coast (average latitude 45°18.5′ N and longitude 12°26.5′ E) on a soft bottom at a depth of 16–22 m. No specific permissions were required for the locations/activities involved in the study, since they were carried out on commercial fishing grounds. The presence of academic staff onboard the fishing vessel was communicated and approved by the Coast Guard of Chioggia (Italy). The study did not involve endangered or protected species. For field experiments, three surveys have been carried out onboard a commercial vessel: April 2010 (spring experiment), July 2010 (summer experiment), and December 2010 (winter experiment). During each experiment, specimens were sampled by means of an otter-trawl during commercial tows of 180 minutes each (n = 4–5 during each field trip) at a speed of 3.7–4.6 km h^−1^ (2.0–2.5 Kts). The water and air temperatures, as well as the salinity during each experiment, are summarised in Table 1, as measured with a minilog DST CTD multiparametric probe (Star Oddi, model S5257) set to record data at 20 second intervals. After retrieving and emptying the net onboard, adult, intermoult [19], live specimens (average carapax length 36.8±5.2 mm; sex ratio 0.40) were randomly collected, within approximately 5–7 minutes of being caught and were immediately used for the physiological stress assessment. Post-branchial hemolymph samples were collected from individuals using a 1 ml syringe inserted into the pericardium. The overall handling and the time of sampling for each individual were kept at minimum (approx. 0.5–1 minutes) to reduce the stress induced by manipulation, although this additional noise cannot be avoided completely. Hemolymph samples were immediately used for pH measurements (see Text S1 for details) and then frozen and kept in liquid nitrogen until arrival in the lab, where they were stored at −20°C until used. Each specimen provided enough hemolymph (approx. 0.5–1.0 ml) to perform analytical measurement of all stress indicators and was used only once. In the summer and autumn experiments (see below), muscle samples were obtained by excising the carapax in the abdominal region at the level of the 3^rd^ and 4^th^ segment; approximately 1 g fresh weight per specimen was excised and immediately frozen in liquid nitrogen.

**Table 1:** Environmental parameters recorded in the field and lab experiments.

EMERSION EXPERIMENTS						
**Field Experiments**	**Salinity (PSU)**		**Water Temp. (°C)**		**Air Temp. (°C)**	**Average Thermal Shock (°C)** *
	Surface	Bottom	Surface	Bottom		
Spring	35	36	15	13	17–19	+5
Summer	30–32	36–37	27–27.5	17	27–28	+10.5
Autumn	34	34–36	12–13	13	12°C	−1
**Lab Experiments**	**Salinity (PSU)**		**Water Temp. (°C)**		**Air Temp. (°C)**	**Average Thermal Shock (°C)** *
Winter	30–35		9–13		8	−3.5
Summer	33–35		16–18		25	+8
Autumn	33–35		8–10		10	+1

In the former case, the values were recorded using a multi-parametric CTD probe during the three field experiments; in the latter case, the values were settled in aquaria.

*Thermal shock (°C) is calculated as T_air_-T_sea bottom_.

For lab experiments, live adult, intermoult [19], specimens (average carapax length 38.2±5.9 mm; sex ratio 0.41) individuals were obtained from commercial fishing and stored in the dark in 70 L of seawater in aquaria (50×34×40 cm) with a 5 cm layer of sand in the bottom. The water was changed every two days. Due to the carnivorous and hunting characteristics of *S. mantis*, farmed specimen were fed *ad libitum* with live individuals of *Atherina boyeri* (big-scale sand smelt). The organisms were starved for 5 days before the emersion experiment. Also in this case, three seasonal experiments were carried out: February 2010 (winter experiment), July 2010 (summer experiment), and November 2010 (autumn experiment). The environmental conditions during farming and emersion are summarised in Table 1. The seawater was adjusted with RED SEA salts to constant salinity (35 PSU) and thermostatted at a temperature close to the sea-bottom temperature as recorded in the same season; for each treatment, individuals were randomly selected from different aquaria (each aquarium contained n = 6 individuals). The pools of individuals used for field and lab experiments were not significantly different in terms of carapax length (one-way ANOVA, p>0.05). Sex ratio recorded on pools subjected to different times of exposure to air/reimmersion in water (see below for experimental details) did not vary significantly between all seasonal (spring, summer, autumn, winter) treatment groups (laboratory, trawled, recovery) (one-way Kruskal-Wallis non-parametric ANOVA, p>0.05).

### Assessment of physiological stress onset; field and lab experiments

In each seasonal field experiment, the pool of individuals used for each experiment was kept in air in plastic containers in the shade. The hemolymph was withdrawn from individuals only once. One group of n = 6 individuals, collected immediately after retrieving the fishing gear, represented the “end of the trawling” or “control” group (“ET”). Hemolymph was withdrawn from other specimens exposed to air to simulate emersion 0.5, 1.0, and 2.0 hours after end of trawling (n = 6 for each time point). The definition of the former ET group as “control” should be treated with caution because these animals were retrieved using the fishing gear and subjected to impact (i.e., physical stress during the tow and pressure and thermal shocks from the sea bottom to deck); thus they represent a control group with respect to the overall further impact due to emersion. This point will be discussed later also in relation to the other “control” group, namely that group used in lab experiments that consisted of farmed organisms not subjected to air emersion. As indicated above, the time delay between hemolymph withdrawal from the first and last individual of a given group (3–6 min) can be considered negligible as compared with the time interval between each experimental group. Lab experiments were carried out with the same emersion times as in the field experiments (0 hours, corresponding to the group of specimens not exposed to air, later on defined as the lab control group, “C”; 0.5, 1, and 2 hours, respectively).

### Recovery from physiological stress imbalance

In order to check that the induced stress did not irreversibly disrupt the homeostatic capability of the specimens, a group of n = 48 individuals was sampled, in the same three seasonal field experiments, after retrieval. They were exposed to air for 0.5 hours, an interval that is consistent with the duration of the sorting process during commercial fishing activities [20] and then were introduced into running seawater to recover from stress. Physiological stress indicators were assessed until 24 hours after re-immersion in tanks filled with running sea-water at the same temperature of the sea bottom (details about the experimental procedure are given in SI1).

### Cumulative post-capture survival

During each seasonal field experiment, the cumulative post-capture survival during emersion was assessed by randomly sampling a pool of individuals (spring, n = 51; summer, n = 82; autumn, n = 35), collected and handled as described above. Mortality was assessed as a function of time and individuals were deemed to be dead when no movement of appendices and no reaction to external stimuli were observed [21]. The cumulative survival rate was also assessed during seasonal laboratory trials on specimens exposed to air at different time intervals.

### Analytical determinations

The hemolymph metabolites (L-lactate, D-glucose, and ammonia) quantified in this study were the same as in [10] while pH was measured in the total hemolymph immediately after sampling. In addition, muscle glycogen concentration was also considered [22]. The methods for quantitative analysis are described in SI1.

### Statistical analyses

A two-way factorial analysis of covariance (ANCOVA) was applied to hemolymph metabolic parameters (D-glucose, L-lactate, ammonia, and pH) and glycogen in order to test the effects of air exposure on farmed and trawled specimens, as well as to compare the early phases of recovery process in individuals re-immersed in water after being trawled and exposed to air. Prior to the analyses, the data were log (*x*+1) transformed in order to achieve a homogeneous variance (tested by means of the Cochrane test); the homogeneity of regressions was also tested [23]. The analyses were set up to assess two fixed factors (season and experimental time, where the latter is the time of emersion or re-immersion in water) and their interactions, testing the effects of a specimen's size (log wet weight; WW) as a covariable. Three different levels for the fixed factor season were considered: spring, summer, and autumn in the field experiments and winter, summer, and autumn in the lab experiments. For glycogen, the seasonal levels were restricted to spring and summer since this parameter was measured only in these seasons. Three to four levels were instead considered for the experimental time factor: C (control), 0.5, 1, and 2 hour exposure to air lab experiment and ET, 0.5 and 1 hour exposure to the air field experiment. When the data did not meet the assumptions of the homogeneity of slopes (glycogen in the lab exposure to air experiment and lactate in the exposure to air field experiments), a temporal trend within each single seasonal experiment was assessed for each parameter by means of the one-way Kruskal-Wallis non-parametric ANOVA. The Mann-Whitney U test [24] was used in order to carry out a pairwise comparison of stress indicators between groups of specimens subjected to treatment at definite experimental conditions within each seasonal experiment. To that end, the following treatment groups were compared: C (laboratory controls), ET (end of trawling, in trawled individuals), EEA (end of exposure to air), 2 and 24 hours (2 and 24 hours after trawled and exposed-to-air individuals were re-immersed in running seawater in the recovery study, respectively; SI1; Table S3).The full time series of data collected in the recovery field experiments was also compared within each seasonal treatment using one-way ANOVA or the Kruskal-Wallis non-parametric ANOVA, when data transformation did not meet the assumptions of the homogeneity of variance (SI1). Seasonal cumulative survival curves were pairwise compared by means of the Gehan-Wilcoxon test [25].

The induction of physiological stress upon trawling and emersion was also analyzed by a factorial approach [26] since it permits the detections of additive, antagonistic, and synergistic effects of different factors. We considered three experimental factors: trawling (Tr), thermal shock (ΔT), and time of exposure to air (Exp) that could affect the five physiological parameters; each factor was set to a (−) and (+) value for factorial analysis. Details about the experimental design and calculation of effects are given in SI1. The set of conditions is summarized in Table S1 and in the design matrix [26] (Table S2). For each factor and level, 6 experimental outputs were available since the same analytical determination was replicated for each of the 6 individuals subjected to a given condition. Second- or third-order interactions were interpreted as deviations from an additive effect when the analysis showed significant departures from the sum of single-stressor effects. In such cases, interactions were interpreted as being synergistic when an increase in stress condition (i.e., an increase in lactate, glucose, ammonia, and H^+^ concentration or a decrease in glycogen) occurred. The opposite approach was applied for the identifying antagonistic effects. It is worth mentioning that for the factorial analysis, the experimental results about acidosis were elaborated in terms of H^+^ concentration rather than pH in order to avoid artifacts due to the logarithmic transformation.

Lab and field data on hemolymph L-lactate, D-glucose, pH, and ammonia were also analyzed by means of Discriminant Canonical Analysis (DCA) on the factors of the Principal Component Analysis (PCA; [27]). Analyses were carried out on 3 sets of independent data: winter and spring, summer, and autumn. DCA was applied with the aim of discriminating between experimental groups (treatments) and identifying the stress indicators that mostly contribute to the discrimination of such groups according to each seasonal lab and field condition. Additional information on DCA analysis is given in SI1.

## Results

### Physiological stress induced by trawling and emersion

**Figure 1:**
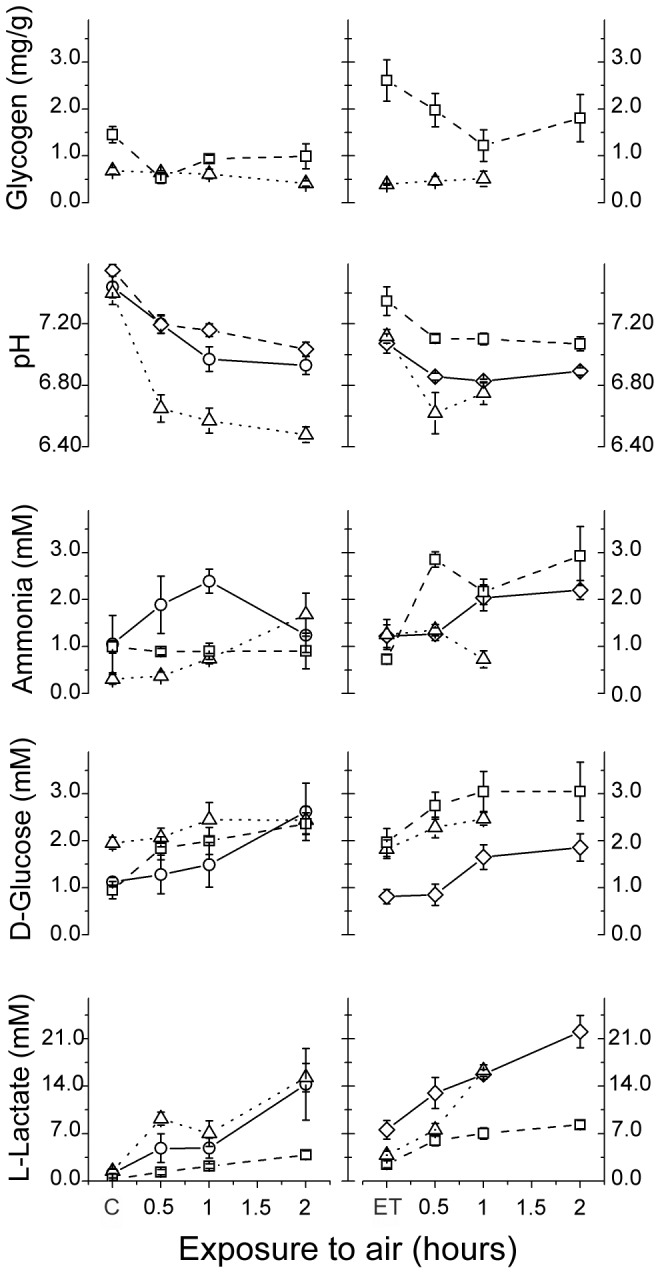
Trends in physiological stress in *Squilla mantis* upon exposure to air. Time dependence of different physiological parameters during the seasonal experiments. Left panels: lab experiments; right panels: field experiments. The symbols denote the different seasons: summer (triangles), autumn (squares), winter (circles), and spring (diamonds). C indicates the time zero (controls) in lab experiments; ET stands for end-of- tow in field experiments.

A first set of seasonal field experiments was carried out on trawled and emersed individuals both in summer and autumn. These experiments were mirrored by parallel emersion experiments carried out on organisms farmed in aquaria under controlled temperature and salinity conditions. While farming conditions could themselves affect some of the measured parameters due to peculiar impacts on the animals (likely diet and energetic turnover), this set of experiments is necessary to assess the physiological response of untrawled individuals to emersion. However, due to climatic and organisms availability constraints, the third field experiment was carried out in spring while the third lab experiment was conducted in winter. As shown in Table 1, the water salinity in all experiments was in the same range (30–37 PSU) and in the field experiments only a small salinity difference was recorded between the bottom and surface water. In the summer and autumn lab experiments, the water and air temperatures were set to match the temperature of bottom water and of air recorded in the field in the corresponding seasonal experiment, meaning that the organisms were exposed to comparable temperatures and thermal shocks (T_air_-T_water_) in the two experimental paradigms. On the contrary, the air temperature in the spring lab experiment was higher than that of the field winter experiment, corresponding to two different thermal shocks. The results relative to the various stress indicators are reported in Figure 1; the left panels report on the lab experiments and the right panels report on the field experiments.

Overall, a clear tendency toward increasing physiological impairment with increasing time of exposure to air was observed both in the lab and field (i.e., post-trawling) experiments; the level of stress was also modulated according to different seasons (see Figure 1 and Tables S3-S5 for a full account of the statistical analysis) while individuals' size had negligible effects on assessed stress indicators.

In the lab experiment, similar increasing trends were observed for the L-lactate in different seasons (2-way ANCOVA; time: p<0.001; season: p<0.001; Table S4). Also, in the trawling experiments and in all seasons, a significant increase in the concentration of this metabolite was observed (Kruskal-Wallis non-parametric ANOVA; spring and autumn: p<0.05; summer: p<0.01). The levels of lactate in the hemolymph of farmed organisms before exposure to air (C; Figure 1, left panel, Table S4) ranged between 0.29±0.17 and 1.47±0.32 mM. In field experiments, higher values were recorded at the end of trawling (2.47±0.51 to 7.57±1.39 mM, ET, Figure 1, right panel, Table S4), although no significant difference was observed between these treatments. Although we do not have data on undisturbed specimens in field experiments, this difference is likely to be attributed to an oxygen debt on the field specimen induced by the trawling and net retrieving operations. Field and lab experiments carried out in the same season with matched temperatures (summer and autumn) showed similar patterns, with higher values recorded when individuals were subjected to higher thermal shock. The increase of lactate hemolymph concentration in spring field experiments (thermal shock +5°C; Table 1) was faster than that observed in winter lab experiments (thermal shock −3.5°C), again attributable to the difference in seasonal temperatures.

In both the lab and field experiments, D-glucose showed significant increasing trends in all seasons (lab experiment, 2-way ANCOVA; time: p<0.001, season: p<0.01; field experiment, 2-way ANCOVA; time: p<0.01; season p<0.001; Figure 1 and Tables S4-S5). No significant differences were recorded between the C and ET groups (Mann-Whitney U test; p>0.05 in all comparisons), showing that the basal levels of glucose within all matched seasons did not differ between farmed and trawled individuals (about 1.0–2.0 mM; Table S3). It is worth noting that in the field experiments, the trends of the summer and autumn data were very similar, in spite of the strong differences in thermal shock experienced by the organisms in the two seasons (+10.5°C and −1°C in the summer and autumn experiment, respectively; Table 1).

The trends of ammonia concentration did not show definite patterns (Figure 1) and in some cases a quite marked variability between individuals was observed. Accordingly, within both lab and field experiments, different significant trends between different seasons were recorded (2-way ANCOVA; season · time, p<0.01; time: and p<0.001, respectively; Tables S4-S5). It is worth noting that the levels of ammonia nitrogen was quite similar both in farmed organisms and specimens trawled in the field (about 0.3–1.3 mM; Table S3). In the lab experiment, an increasing trend was observed in summer, while in autumn the values were almost stable. The overall peak in ammonia was observed in the winter experiment after 1 hour of exposure to air, although in the following treatment (2 hours of exposure to air) values sharply declined. On the contrary, in the trawling experiments, exposure to air induced an increase in ammonia concentration both in autumn and spring, while in the summer a decrease in this parameter was recorded 1 hour after the end of trawling.

The pH showed an immediate decrease during exposure to air both in the lab and field experiments, as hemolymph H^+^ increases due to the disturbance (Figure 1, Tables S4-S5). Decreasing pH trends significantly differed among seasons in the lab experiments (2-way ANCOVA, season · time, p<0.001; time, p<0.001). Furthermore, pH proved to be influenced by individuals' sizes (2-way ANCOVA, log WW, p<0.01), showing a positive correlation with size. On the contrary, the time dependency of pH did not differ significantly between seasons in the trawling experiments (2-way ANCOVA, time: p<0.001; season: p<0.001). This effect might be attributable to the lower pH average values at the beginning of the field experiments (about 7.1–7.3) compared with the lab experiments (about 7.4–7.5; Table S3).

The content of glycogen, the typical energy storage of muscles, showed a significant temporal pattern (Figure 1, Tables S4-S5) only in autumn (Kruskal-Wallis non-parametric ANOVA, p<0.05) in the lab experiments, whereas different trends were observed between summer and autumn in the field experiment (2-way ANCOVA, season · time, p<0.05). In both experimental paradigms, in autumn a decrease in the glycogen was observed during emersion, although the basal values recorded in the field experiment had higher, but not significant, average values (about 2.6 mg/g and 1.5 mg/g, respectively; Mann-Whitney U test, p>0.05, Table S3) compared with the lab experiment. Farmed animals exhibited a much lower content of glycogen in their muscles, as compared with field specimens. In the summer experiment, the starting values of glycogen of both lab and field experiments were very low and similar in the two experimental paradigms. Furthermore, the trends as a function of time after emersion almost overlapped in this season.

### Recovery from physiological imbalance

The time dependence of the recovery from physiological stress was studied. Table 2 summarizes the environmental conditions of the three seasonal recovery experiments. As detailed in SI2, Figure S1 and Tables S6-S7, all stress indicators returned to the levels of the undisturbed specimens, indicating that the disturbance induced in our experimental paradigm does not irreversibly disrupt the homeostatic capacity of the organisms.

**Table 2:** Environmental parameters recorded during recovery in the field experiments.

RECOVERY EXPERIMENTS				
Field Experiments	Salinity (PSU)	Water Temp. (°C)	Air Temp. (°C)	Average Thermal Shock (°C)**
Spring	33–35	13	17	+4
Summer	33–35	12–15*	27	+13.5
Autumn	33–35	13	13	0

The values were recorded using a multi-parametric CTD probe during the three field experiments.

* This temperature was set by a circulating cooling system.

** This thermal shock (°C) is calculated as T_air_-T_water tank_.

### Factorial analysis of physiological stress induced by trawling, emersion and thermal shock

**Figure 2:**
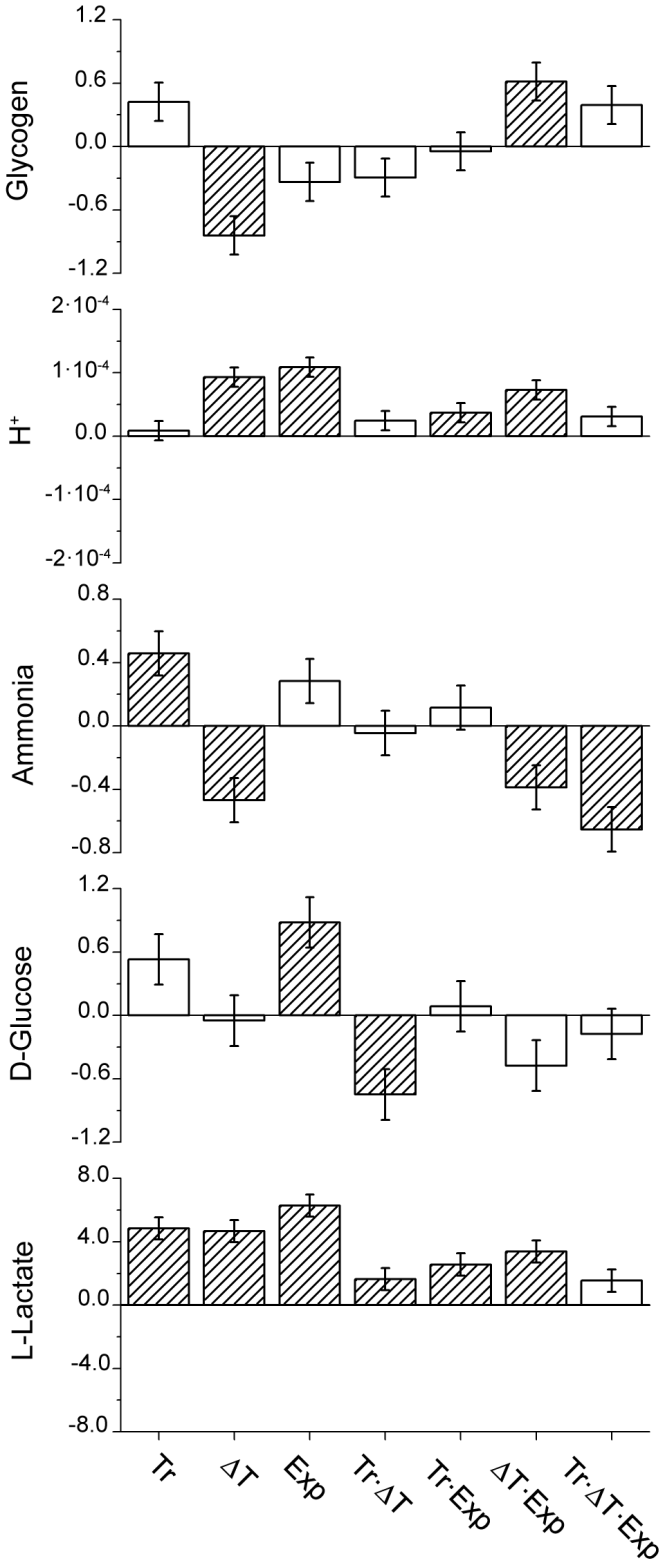
Results of the factorial analysis of physiological stress on *Squilla mantis*. The factors are: “trawling” (Tr), “thermal shock” (ΔT: T_air_ - T_water_), and “exposure time” (Exp) at two levels (+) and (−), as described in the Materials and Methods section. The bars refer to the “main effects” of the single factors – E(Tr), E(ΔT), E(Exp) – and to the effects of their two-levels interactions – E(Tr · ΔT), E(Tr · Exp), and E(ΔT · Exp) – and the three-level interaction E(Tr · ΔT · Exp), calculated according to Equations 1–7 of SI1. The experimental data are from Figure 1, extracted according to the scheme of Tables SI1 and SI2. Dashed bars are the significant effects at p<0.05 or better; empty bars are the non-significant effects. The values on the *y*-axis have the same dimension as the corresponding parameter: mM (in the case of L-lactate, D-glucose, ammonia nitrogen, and H^+^) or mg g^−1^ (in the case of glycogen).

The results of the factorial analysis on the different indicators are summarised in Figure 2 in the form of histograms where the bars refer to the “main effects” of the single factors – E(Tr), E(ΔT), and E(Exp) – and the effects of their two-levels interactions – E(Tr · ΔT), E(Tr · Exp), and E(ΔT · Exp) – and three-levels interaction E(Tr · ΔT · Exp). The numerical values quantifying the two levels and three levels interactions are summarised in Figures S3 and S4, respectively. These figures report the concentrations of each hemolymph indicator when each distinct interacting factor changes between the (−) and (+) conditions.

The main effects of factors should be individually interpreted only when there are no significant interactions among factors; when interactions are found to be significant, the different factors should be discussed jointly [26]. In the case of L-lactate, significant synergistic effects have been obtained in all of the two-levels interactions. This pattern shows that the significant increase observed in this parameter due to the effect of each single factor (trawling, thermal shocks, and time of exposure) is further exacerbated when each factor interacts with another although the three-level interaction is not significant. Trawling and thermal shock (Tr · ΔT), when jointly considered, showed an antagonistic effect on D-glucose although, as single factors, they did not significantly affect the levels of that parameter. It is worth noting that, on the contrary, Exp affected D-glucose, increasing significantly its concentration. The total ammonia nitrogen concentration showed the most complex pattern, since overall the third-order interaction (Tr · ΔT · Exp) was significant and negative, pointing to an antagonistic effect. When considering the two-level interactions, the joint effect of thermal shock and time of exposure (ΔT · Exp) was, again, antagonistic and thus in the same “direction” as the single effect of thermal shock, which was significant. Also, trawling had a significant effect on ammonium, increasing its concentration significantly. The fact that the interaction between trawling and thermal shock was not significant points to a compensation between the two opposite, and significant, effects of each single factor. When considering H^+^, both thermal shock and exposure to air (Tr · Exp) and trawling and exposure to air (ΔT · Exp) showed a synergistic effect, with a higher effect for the first two-level interaction. Glycogen showed an antagonistic effect in the interaction of thermal shock and exposure time factors (ΔT · Exp), since a significant increase in the parameter was found. It is worth noting that only thermal shock negatively affected (i.e., reduced) the glycogen tissue content.

### Cumulative survival in lab and field experiments

**Figure 3:**
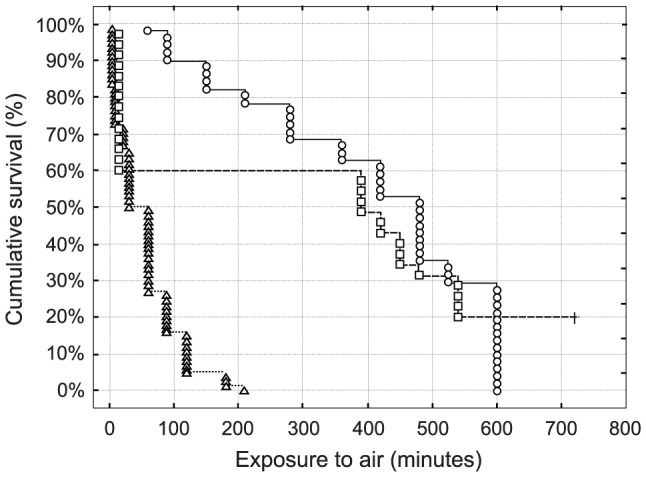
Cumulative percentage survival of *Squilla mantis* recorded after trawling during exposure to air. The symbols denote the different seasons: summer (triangles), autumn (squares), and winter (circles). It is worth noting that in lab experiments the cumulative survival rate was 100% after 120 minutes of exposure to air, regardless of the season and the thermal shock.

The cumulative survival percentage of specimens was studied as a function of time to provide a measure of the lethal effects of air exposure over time and their temperature/seasonal dependence (Figure 3). In all lab experiments, the cumulative survival observed within 120 minutes was 100% regardless the seasonal and thermal shock the specimens were subjected to (data not shown). On the contrary, during field experiments, high mortality rates were recorded, showing different patterns according to season. In the summer experiment, characterized by strongly positive thermal shocks (Table 1); 50% of specimen were dead 20 minutes after emersion. In contrast, in the spring and autumn experiments, when the thermal shock was more limited or negligible (Table 1), 50% mortality was recorded at about 7 hours after emersion. Accordingly, cumulative survival curves in summer differed significantly between spring and autumn (Gehan-Wilcoxon test, p<0.001 in both comparisons). It is worth noting that post-trawling cumulative survival in spring and autumn did not differ significantly (Gehan-Wilcoxon test, p>0.05), although a higher mortality rate was observed at the begin of the autumn experiment.

### Multivariate analysis

DCA showed that the stress indicator patterns contributed significantly to the discrimination of groups (treatments) in all considered seasons (p<0.001 in all seasons).

The first two discriminant canonical functions explained, respectively, 70.3% (winter-spring), 70.2% (summer), and 68.0% (autumn) of the overall variability, confirming the good quality of each representation. In all considered seasons, the pH had a significant negative correlation (p<0.05) with the other indicators, while a significant positive correlation (p<0.05) was found among glucose, ammonia, and lactate in autumn and winter-spring. Moreover, in all considered seasons, the pH was significantly correlated with the first discriminant function while the other stress indicators were mainly correlated with the second discriminant function. DCA plots are reported in Figures S5-S7. The full description of the multivariate analysis results is given in Text S2.

Overall, trawling and exposure to air produced the most relevant effects on *S. mantis* individuals and this effect was more marked when individuals were subjected to high thermal shock (i.e., in summer).

## Discussion

Thermal shock, exposure to air, and trawling are stressors that are known to induce physiological disruption in marine bycaught species [12]. The ultimate effect of stress is modulated by the ecophysiological features of disturbed species, as well as by the strength (i.e., level) and persistency (i.e., time of exposure) of stressors, and may lead to large mortality rates.

*Squilla mantis* was selected as a guide species since it is a widespread stomatopod in the Mediterranean Sea and it inhabits even relatively shallow waters (i.e., around 20 m) where the variability of environmental features like seawater temperature and salinity can be intermediate, thus pointing to a medium adaptation to environmental fluctuations. Moreover, this species is subjected to commercial fishing as it is targeted by trawling or discarded as non-commercial species in different Mediterranean Sea areas.

As stress indicators, we focused on D-glucose, L-lactate, ammonia, pH, and muscle glycogen concentration since they are able to trace homeostasis disruption and adaptive/maladaptive responses [11] in relation to different stressors, Accordingly, we carried out a set of experiments to trace the dynamics of the onset of physiological stress impairment to understand and describe the physiological processes that undermine an individual's homeostasis as well as to test the role of different stressors. This approach was mirrored by a recovery study that shed light on the capability of stressed pools of individuals to recover from physiological imbalances.

The mortality tests clearly showed that exposure to air does not induce short-term (i.e., up to two hours) mortality in control individuals (stored in aquaria), the thermal shock they were subjected to notwithstanding. On the contrary, field experiments showed that individuals exposed to air after trawling are subject to increasing mortality over time and that the highest cumulative mortality was correlated with a higher thermal shock and time of exposure to air. Our result also agrees with those obtained for *S. mantis* in the upper Northern Adriatic Sea, where a 100% mortality rate was recorded for trawled individuals in an autumn experimental trawl although the seasonal pattern in post-trawl mortality was not assessed in the study [17]. The seasonal pattern in short-term mortality recorded in our study is also consistent with the results of other studies conducted on the portunid crab *Liocarcinus depurator* in the same area that pointed to marked seasonal effects on mortality rates and physiological impairment, determined by the different degrees of thermal shock individuals were exposed to upon after trawling [9]–[10]. Benthic crustaceans often live under a thermocline and halocline and thus inhabit relatively stable environments and are virtually unable to cope with emersion; furthermore, thermal and osmotic shocks might add to a respiratory imbalance. These disturbances generally produce cumulative consequences on organisms and usually result in high mortality rates [28]–[29], [21]. It is worth noting that in our experimental paradigm sea surface salinity was almost close to sea-bottom conditions, thus pointing to a negligible effect of such a factor influencing *S. mantis* mortality. This factor might play a relevant role in *S. mantis* stress and mortality, in agreement to what has been shown in laboratory trials in *Nephrops norvegicus* [30]. However, experimental trials of the changes in physiological parameters due to exposure to low-salinity seawater (Text S2; Figure S2) showed that *S. mantis* experienced readable physiological changes when the salinity was set at about 20–25 PSU with a sharp decrease in osmolality; the increase in glucose concentration at 30 PSU or lower levels might be related to the mobilization of energy to cope with osmotic processes. Thus, while in our experimental trials the seawater salinity was almost close to sea-bottom conditions, this factor should be taken into account in particular when individuals are trawled close to river mouths.

It is worth noting that short-term survival experiments might underestimate the ultimate effects of discarded specimens [21]. Post-capture mortality may occur until 21 days after trawling, owing to the presence of sub-lethal damages incurred by trawling and following sorting operations (e.g., wounds, loss in appendages, internal damages). In the pool of individuals used in our experimental studies, only a small portion (less than 10%) showed the loss of one appendage, while the presence of specimens with a higher frequency of visible physical damages was negligible (less than 1%). Thus, this factor can be considered negligible in shaping the patterns of stress we recorded, while other wounds (not visible by an external inspection) might have played a more relevant role that cannot, however, be assessed given the experimental conditions.

### Physiological stress onset and recovery

Thermal shock, fishing capture, and exposure to air may impact the organism physiology by inducing: i) an increase of energy expenditure due to an increase in metabolic rates, ii) an increase of energy expenditure due to behavioural responses and fatigue, and iii) the collapse of the gills under emersion and on-deck storage. Overall, a clear tendency toward physiological disruption both in emersed and trawled and emersed individuals was recorded by physiological metabolites. Furthermore, seasonality in the field and lab experiments influenced the degree of homeostasis disruption in the experimental organisms, pointing to a major role of temperature shock as a driver able to modulate physiological impairment onset.

The observed increase of hemolymph levels of lactate was expected due to the internal hypoxia resulting from both the mechanical stress during trawling and the gill collapse upon emersion. The increase of lactate concentration was faster at higher temperatures, in agreement with the temperature enhancement of metabolic rate for heterothermal organisms. This consideration may apply also to explain the lower lactate levels recorded in autumn, when both the thermal shock and sea bottom temperatures (Table I) were the lowest in our field experimental paradigm. The levels of glucose are expected to be related to those of lactate since glucose represents the substrate to sustain cytoplasmic glycolysis and its levels are homeostatically controlled in crustacea by a variety of mechanisms including catecholamines and the Crustacean Hyperglycemic Hormone (cHH) [31]. These chemical messengers are released by neurosecretory structures in the eyestalks of stalk-eyed decapod crustaceans such as *S. mantis* [32] as responses to environmental stressors. Furthermore, the increase in glycolysis rate after air exposure is facilitated possibly by cHH, which increases substrate availability by glycogen degradation [33]–[34]. In crustacea, gills are important also for ammonia and ammonium salt excretion. This process is sustained by a Na^+^/K^+^ pump and V-type H^+^ pump with the involvement of Na^+^/NH_4_^+^ and NKCC transporters [35]–[38]. In case of emersion, the collapse of gills impairs these transports and an accumulation of ammonia nitrogen is expected. Gill collapse is responsible also for the impairment of carbon dioxide ventilation and, together with the accumulation of lactic acid, is responsible for hemolymph acidosis. Furthermore, the mechanical exercise increases acidosis due to an accelerated respiration, as it occurs in the case of trawled specimens exhibiting lower pH levels as compared with farmed organisms (Figure 1, C and ET in the lab and field experiments, respectively). The acidosis appears to be more marked when the thermal shocks are higher in the summer lab experiment and the spring and summer field experiments. The thermal change can alter the acid–base status in the lobster *Homarus americanus* over a timescale of minutes. Acute increases in temperature trigger metabolic acidosis of the hemolymph. Homeostatic mechanisms involve the strength and frequency of the heartbeat that are modulated by changes in pH [39]. These cardiac output variations increase the gill gas exchange efficiency for immersed organisms; respiration compensates for the temperature effect but is likely not to be effective in the case of emersed aquatic organisms where gill ventilation is impaired. The content of glycogen, the typical energy storage of muscles, is found to be markedly different between farmed and wild organism used for the autumn experiments. In particular, farmed animals exhibited a much lower content of glycogen in their muscles compared with field specimens. These differences, found in the same seasonal experiment, can be attributed to a depletion of glycogen in the muscles as a result of farming. Therefore, only the field experiment provides reliable differences as a function of time and between the two seasonal replicates. Glycogen is found to decrease as a function of time of emersion, in agreement with the onset of respiratory and energetic stress. Glycogen depletion during hypoxia was also reported in the case of crayfishes [40]. In the summer field experiment, the levels of glycogen were very low both in the lab and field experiments and agreed in the two experimental paradigms. Furthermore, the trends as a function of time after emersion almost overlapped. Glycogen does not represent, therefore, a significant energy supply and its levels remain constant as a function of time.

It is worth noting that process of physiological disruption induced by thermal shock, trawling, and air exposure were shown to be reversible, since recovery experiments confirmed that within 24 hours after being trawled and exposed to air, *S. mantis* was found to be able to recover from physiological imbalance. The timing of recovery, is, however, not immediate after re-immersion in water, since stress can still increase up to 2–4 hours afterward (as in the case of lactate), while, at the same time, the mobilization of glucose to recover from the impairment is still high. These results are consistent with other studies showing that physiological parameters can be recovered within one day after caught individuals are returned to the sea [8], [10].

### The role of different factors in shaping *Squilla mantis* physiological disruption

The factorial analysis allows us to shed light on the relative role of different factors on inducing stress in *S. mantis*. We applied an additive model for the analyses of our factorial study since this model is the most suitable for the analysis of indicators related to physiological processes [41]. However, the interpretation of our factorial study should be carried out taking into account some relevant caveats. First of all, a factorial study, *per se*, needs to analyze the definition of the levels of stressors. In our case, we focused on the presence/absence of emersion to air, negligible vs. 10°C positive thermal shock, and the absence of exposure to air or exposure to air lasting for a duration of 1 hour. These levels were defined according to the field and lab conditions encountered during our experiments; in particular, a thermal shock of about 10°C is the maximum observed in our seasonal studies, while 1 hour of exposure to air may determine severe mortality rates, especially during summer, on trawled and emersed individuals. Another caveat is related to the fact that synergistic or antagonistic effects of stressors have to be interpreted taking into account that stress indicators are modulated by dynamic physiological processes that may interact, resulting in non-linear responses [5], [42]–[43]. Moreover, the expression of metabolite concentration is necessarily characterized by limits (upper and lower) related to physiological processes, metabolic rates, and individual resistance, beyond which an increase (or decrease) in metabolites is physiologically not possible [44]. Indeed, the survival experiments showed that the most stressful conditions considered in our factorial study (i.e., individuals being subjected to thermal shock, trawled, and exposed to air) are consistent with the highest mortality rates encountered in our field experiments, highlighting that hemolymph sampling was carried out on the group of individuals that survived the overall cumulative stress, thus representing a sort of “censored” experiment. Accordingly, it could be expected that the overall interaction of the three factors does not result in synergistic effects in all considered parameters while many second-order significant synergistic interactions were detected. For instance, considering lactate, all two-level interactions were significant, pointing to a further increase in lactate concentration when two stressors acted together. In particular, the combination of thermal shock and time of exposure to air induced the most severe effects. This result could be interpreted considering that the thermal shock induces an increase in metabolic rate while exposure to air leads to internal hypoxia, whose extent increases with the duration of air exposure [45]–[47]. In contrast, glucose mobilization was positively affected only by the time of air exposure, as a single factor, showing that at the considered levels of stressors the physiological response in mobilizing energy has a time delay, possibly related to the release of cHH, and time is needed to identify changes in its concentration in hemolymph [48]. This process of adaptive response [11] can also be detected when considering the time dependency of this parameter in the physiological onset of stress experiments. It is worth noting that the combined effect of trawling and thermal shock showed an antagonistic effect (while these factors, when considered as single factors, had no statistical relevance).

A synergistic effect between thermal shock and time of exposure to air can be seen also when considering H^+^, since a further increase in H^+^ is revealed when compared with the single effect of each stressor. This significant result also applies to the interaction between trawling and time, although with a lower magnitude; moreover, it suggests that the time of exposure influences the degree of acidosis shown by *S. mantis*. The lack of a significant third-order interaction that shows the presence of an additive effect when all stressors act together could be interpreted considering the trend in pH over time: the tendency of acidosis levels to converge to a minimum value around 6.8, which could be thus considered the physiological limit of expression of pH.

Pertaining to glycogen, the interpretation of the factorial study is less straightforward and this result might be ascribed, as already shown, to the different conditions observed in farmed and field-collected individuals.

Finally, ammonia showed a third-order antagonistic interaction among factors. This result indicates that overall effect of impairment in ammonia excretion, at the experimental level where the factors were assessed, cannot be easily predicted and that the reduction in ammonia concentration observed due to the single effect of thermal shock effect prevails over other factors. This result, which differs from what we expected, can be rationalized considering that a further increase in ammonia concentration was seen in trawled individuals, when the time of exposure to air was extended to 2 hours, pointing to a delayed accumulation of this metabolite in *S. mantis*. A similar trend of delayed increase in ammonia concentration during emersion was observed in winter, for instance, in the decapod *Maia squinado* [49]. Observations made in the Northern Adriatic Sea on trawled and emersed individuals of the portunid crab *Liocarcinus depurator* showed different patterns in ammonia modulation according to season: during summer, ammonia concentration was high and did not increase significantly over time while in winter experiments, when lower hemolymph ammonia concentrations were recorded, a significant positive trend during emersion was detected [10].

### *Squilla mantis* physiological limits vs. stressors critical limits

The multivariate analysis of lab and field data on stress indicators and the outcomes of survival test allows us to provide an integrated interpretation of the displacement from homeostasis conditions (control groups) and the following recovery from physiological imbalance, that follows a clear path. Stress increases as individuals are exposed to air in lab conditions, but the degree of physiological imbalance is further worsened in individuals caught by trawling. Moreover, the time of exposure is a key factor in driving the dynamic modulation of both adaptive (glucose mobilization) and maladaptive (i.e., a decrease in pH, a decrease in lactate, and an increase in ammonia) physiological responses. The most stressful conditions are those encountered when the three factors act together, in particular in individuals subjected to trawling and to at least 30-60 minutes after emersion.

Species' thermal tolerance and critical limits have been studied to ascertain the role of climate change on shaping the distribution of marine species [50]. In this context, it has been shown that the interaction with low CO_2_ and hypoxia may result in metabolic depression, worsening hypoxemia and causing a narrowing of thermal performance windows, thus prematurely leading the organism to the limits of its thermal acclimation capacity [47]. This process might be similar to the process that determined the observed mortality in *S. mantis* when thermal shock was added to trawling and exposure to air: the physiological tolerance window resulting from the interactions of the three factors is restricted to result, at least in our study, in large mortality rates.

However, a generalization of the outcomes of our study to other crustaceans should treated with caution since it is important to take into account species' physiological and environmental adaptations. Indeed, as demonstrated in the land snail *Cornu aspersum* in reference to thermal tolerance and critical limits, these parameters vary according to adaptations to the local environment [51]. Since in our study area environmental fluctuations may be considered intermediate (according to the limited depth), *S. mantis* specimens or other species distributed in more stable environments (i.e., at larger depths) might show a higher sensitivity compared to specimens/species that inhabit more variable environments (e.g., intertidal areas) as shown for instance by crabs that inhabits high intertidal zones [52].

Our factorial study showed that the multiple interactions of the three stressors do not act synergistically on the modulation of physiological parameters but have additive effects, while second-order interactions resulted in synergistic effects. Indeed, pH stabilizes at values of about 6.8, D-glucose stabilizes at about 3.0 mM, and ammonia stabilizes at about 3.0 mM; only lactate increased further, at 2 hours of exposure to air, up to 21.5 mM. These values are probably the maximum physiological limits that can be sustained by individuals and are reached when individuals also show an increased mortality rate.

Accordingly, it is possible to rationalize the inherent difficulties in using physiological indicators to predict mortality [11]: when critical limits are reached, the dynamic modulation of metabolites may also reach an almost-steady state. Mortality occurs and data collected on survivors can only show the pattern of homeostasis disruption in individuals that are still able to sustain the stress they are subjected to, also justifying the loss in synergies observed in our factorial study.

## Conclusions

This work represents an integration of lab and field studies of the physiological imbalance induced by three different factors: exposure to air, thermal shock, and trawling. Overall, the three factors did not act synergistically at the physiological level, while synergistic effects were found when two factors acted together. This result can be interpreted in light of the dynamic physiological adaptive and maladaptive processes that are determined by the stressors. Stress induction results in a dynamic response that may encounter physiological limits when high stress is sustained. Overall, when critical limits are encountered, mortality occurs and physiological parameters reflect the response of the survivors. In light of our survival studies, thermal shock and exposure to air primarily affect the mortality of *S. mantis* only on trawled individuals, while lab-stored individuals did not show any mortality during exposure to air until after 2 hours. Our study also highlights that in order to reduce post-trawl mortality in discarded crustaceans, it is necessary to reduce the duration of the sorting operations (i.e., exposure to air), avoid fishing operations where higher positive thermal shocks are expected (i.e., in temperate seas or during summer), and use less stressful fishing gear like static gears.

## Supporting Information

Figure S1 **Recovery from physiological stress in** ***Squilla mantis*** **upon immersion in water after air exposure.** Time dependence of different physiological parameters during the seasonal experiments. Left panels: overall results; right panels: magnification of the first two hours' recovery. The symbols denote the different seasons: summer (triangles), autumn (squares), spring and (diamonds). EEA stands for End of Exposure to Air.
(TIF)

Figure S2 **Effect of salinity changes on hemolymph parameters in** ***Squilla mantis*** **.** Top Panel: osmolarity; Bottom panel: concentration of lactate (white bars), glucose (black bars), and ammonia (gray bars); the values refer to the left *y*-axis. The pH values (hatched bars) are also shown; the values refer to the right *y*-axis. In the pictures, the mean values ± the standard deviation are reported.
(TIF)

Figure S3 **Factorial analysis: numerical values of significant effects of two-factor interactions on** ***Squilla mantis*** **.** The factors are: “trawling” (Tr), “thermal shock” (ΔT: T_air_ - T_water_), and “exposure time” (Exp) at two levels (+) and (−), as described in the Materials and Methods section.
(TIF)

Figure S4 **Results of the factorial analysis: numerical values of significant effects of three-factor interactions (ammonia).** The factors are: “trawling” (Tr), “thermal shock” (ΔT: T_air_ - T_water_), and “exposure time” (Exp) at two levels (+) and (−), as described in the Materials and Methods section.
(TIF)

Figure S5 **DCA plots applied considering stress indicators in winter and spring experiments.** The analysis considered stress-indicator data (glucose, lactate, ammonia, and pH) according to two seasonal datasets (winter: W; spring: S). The figure is a plot composed of i) a plot of the canonical weights (top left), ii) a plot of correlations between discriminant variables and functions (bottom left), and iii) a plot of the canonical scores with ellipses and gravity. C  =  laboratory control group; EL  =  emersed groups in lab conditions; ET  =  trawled groups; EEA  =  end of exposure to air after trawling in the recovery study; R  =  individuals exposed to air and re-immersed in tanks with running seawater. 0, 0.5, 1, 2, 4, 8, 12, and 24: time after exposure to air or re-immersion in tanks with running seawater, in hours.
(TIF)

Figure S6 **DCA plots applied considering stress indicators in summer experiments.** The analysis considered stress-indicator data (glucose, lactate, ammonia, and pH) according to summer seasonal dataset (S). The figure is a plot composed of i) a plot of the canonical weights (top left), ii) a plot of correlations between discriminant variables and functions (bottom left), and iii) a plot of the canonical scores with ellipses and gravity. C  =  laboratory control group; EL  =  emersed groups in lab conditions; ET  =  trawled groups; EEA  =  end of exposure to air after trawling in the recovery study; R  =  individuals exposed to air and re-immersed in tanks with running seawater. 0, 0.5, 1, 2: hours after exposure to air or re-immersion in tanks with running seawater, in hours.
(TIF)

Figure S7 **DCA plots applied considering stress indicators in autumn experiments.** The analysis considered stress-indicator data (glucose, lactate, ammonia, and pH) according to the autumn seasonal dataset (A).The figure is a plot composed of i) a plot of the canonical weights (top left), ii) a plot of correlations between discriminant variables and functions (bottom left), and iii) a plot of the canonical scores with ellipses and gravity. C  =  laboratory control group; EL  =  emersed groups in lab conditions; ET  =  trawled groups; EEA  =  end of exposure to air after trawling in the recovery study; R  =  individuals exposed to air and re-immersed in tanks with running seawater. 0, 0.5, 1, 24, 8, 12, and 24: time after exposure to air or re-immersion in tanks with running seawater, in hours.
(TIF)

Table S1 **Factorial experiment: factors and levels.** Summary of experimental conditions (from Table 1) used in the factorial analysis of the effects on physiological parameters of the three factors at two levels. ^a^ΔT =  T_air_-T_water_. ^b^ -1/+1°C in field and lab experiments, respectively. ^c^ +8.0/+10.5°C in lab and field experiments, respectively.
(DOC)

Table S2 **Factorial experiment: design matrix.** Summary of the combination of factors and levels for the different 2^3^ experimental conditions used in the factorial analysis. Effects refer to the physiological parameter investigated: L-lactate, D-glucose, the total ammonium nitrogen hemolymph concentration, hemolymph pH, and muscle glycogen concentration. The signs identifying the level of each factor in a given condition are shown in each box. To calculate the effect E, the results R_i_ for each condition C_i_ are added, each one with a sign in correspondence of a given column. ^a^Experimental conditions as in Figure 1. ^b^Results of analytical determinations of physiological parameters: concentrations of L-lactate, D-glucose, ammonia nitrogen, H^+^ (mM), and concentration of glycogen (in mg g^−1^). ^c^ *n* is the number of the equation described in Text SI (1. Material and Methods) used to calculate the given effect.
(DOC)

Table S3 **Experimental values observed in** ***Squilla mantis*** **at different treatment levels.** Organisms in aquaria in lab experiment (C); organisms at the end of trawling in field experiments (ET); trawled organisms in field experiments after exposure to air (0.5 hours, EEA); organisms after 2 and 24 hours recovery in water in the field experiments. Values are expressed as a mean ± standard error (n = 6 individuals for each treatment). Significant pairwise comparisons are highlighted between treatments and are shown at the top with bold characters (Mann-Whitney U test, p<0.05).
(DOC)

Table S4 **Exposure to air lab experiment (0–2 hours): 2-Way ANCOVA results.** Significant effects are highlighted in bold.
(DOC)

Table S5 **Exposure to air field experiment (0–2 hours): 2-Way ANCOVA results.** Significant effects are highlighted in bold.
(DOC)

Table S6 **Early phase (0–2 hours) of the recovery process: 2-Way ANCOVA results.** Significant effects are highlighted in bold.
(DOC)

Table S7 **Whole recovery experiment: 1-Way ANOVA results.** Significant effects are highlighted in bold.
(DOC)

Text S1 **Supporting Information for Materials and Methods.**
(DOC)

Text S2 **Supporting Information for Results.**
(DOC)
