# Supplementary figures and images for: Synergistic and Antagonistic Effects of Thermal Shock, Air Exposure, and Fishing Capture on the Physiological Stress of Squilla mantis (Stomatopoda)
Source: PLoS One. 2014 Aug 18;9(8):e105060. doi: 10.1371/journal.pone.0105060 (PMC4136847; doi:10.1371/journal.pone.0105060)

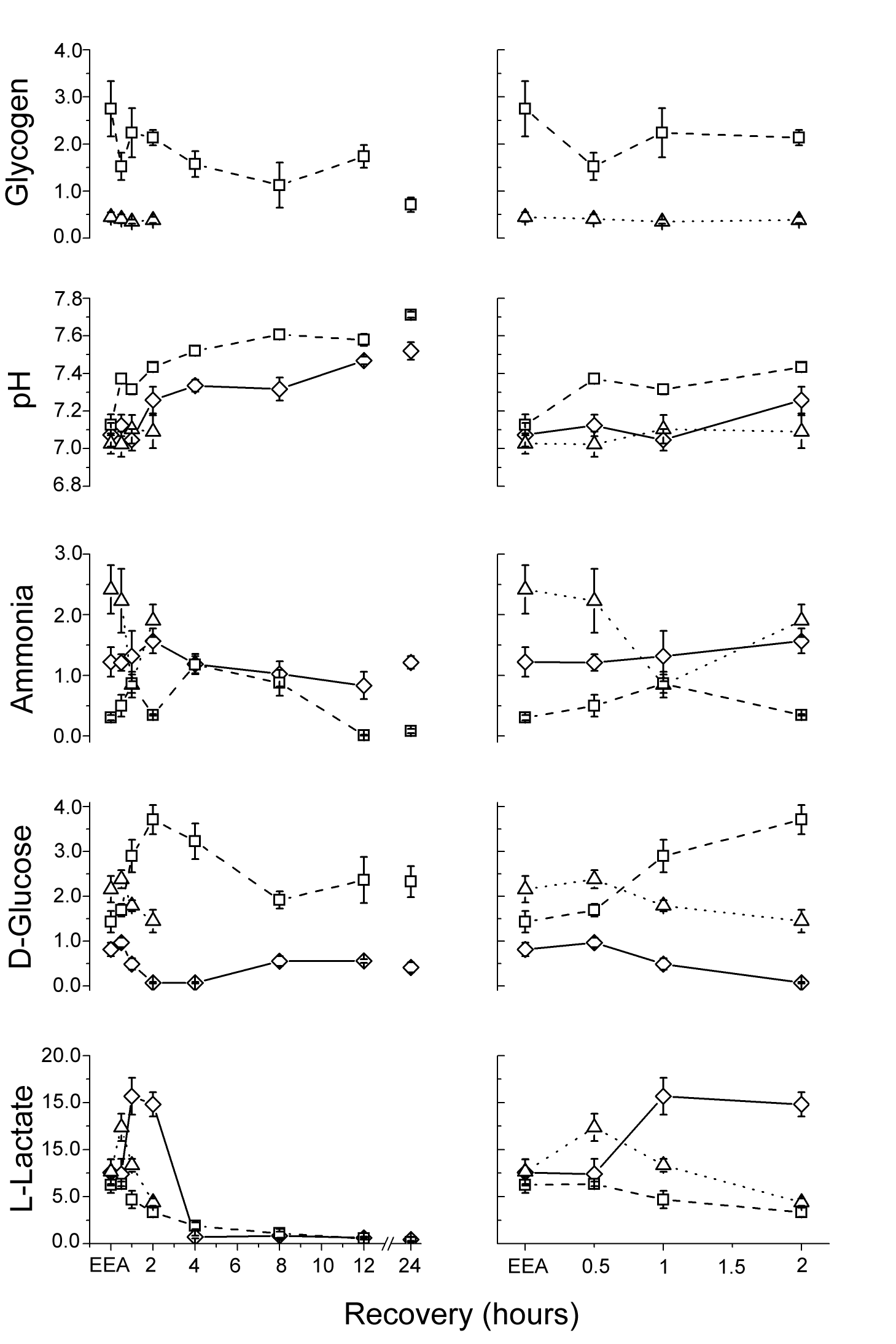

Supplement: Figure S1 — Recovery from physiological stress in Squilla mantis upon immersion in water after air exposure. Time dependence of different physiological parameters during the seasonal experiments. Left panels: overall results; right panels: magnification of the first two hours' recovery. The symbols denote the different seasons: summer (triangles), autumn (squares), spring and (diamonds). EEA stands for End of Exposure to Air. (TIF) [file pone.0105060.s001.tif]

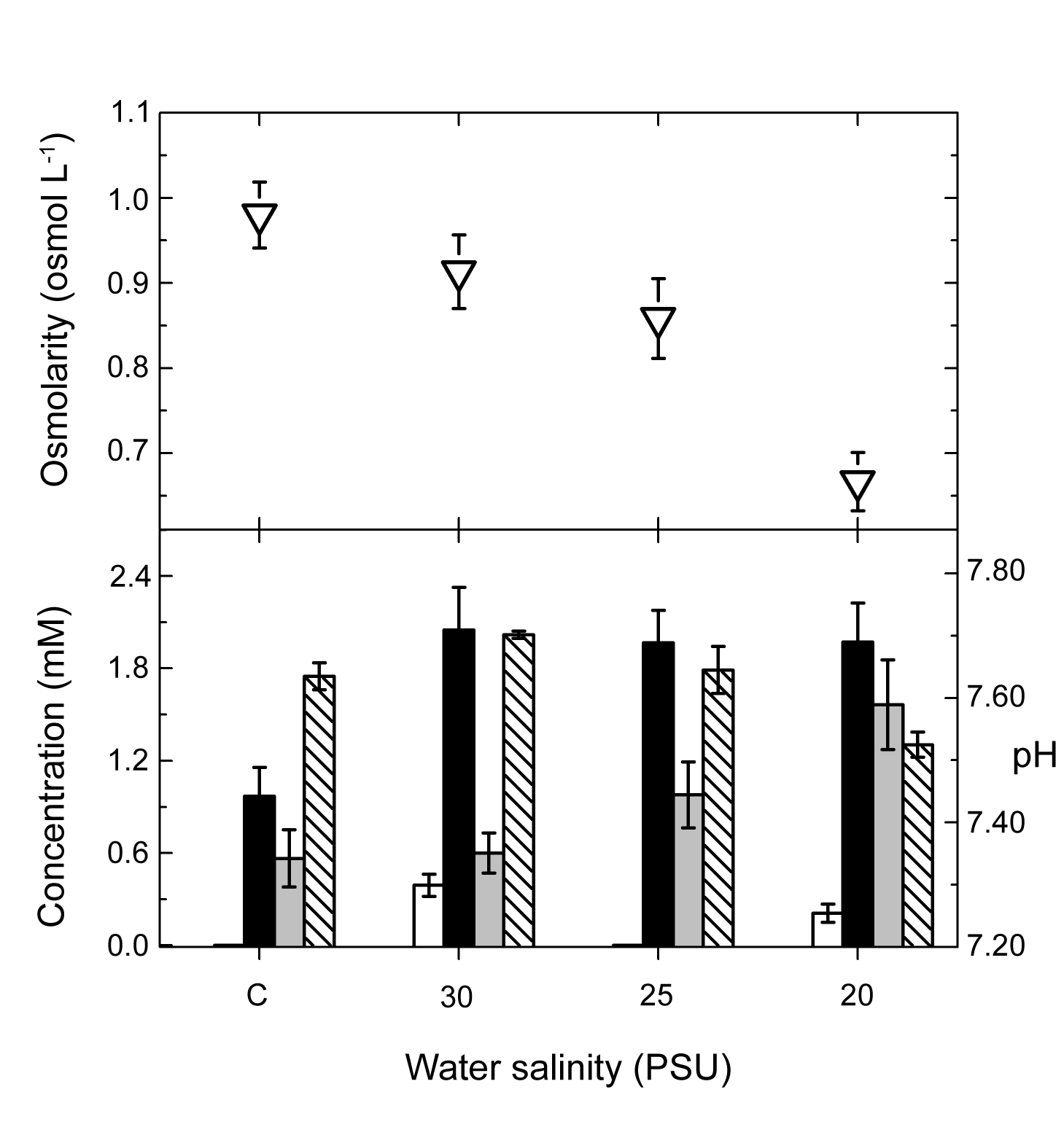

Supplement: Figure S2 — Effect of salinity changes on hemolymph parameters in Squilla mantis . Top Panel: osmolarity; Bottom panel: concentration of lactate (white bars), glucose (black bars), and ammonia (gray bars); the values refer to the left y-axis. The pH values (hatched bars) are also shown; the values refer to the right y-axis. In the pictures, the mean values ± the standard deviation are reported. (TIF) [file pone.0105060.s002.tif]

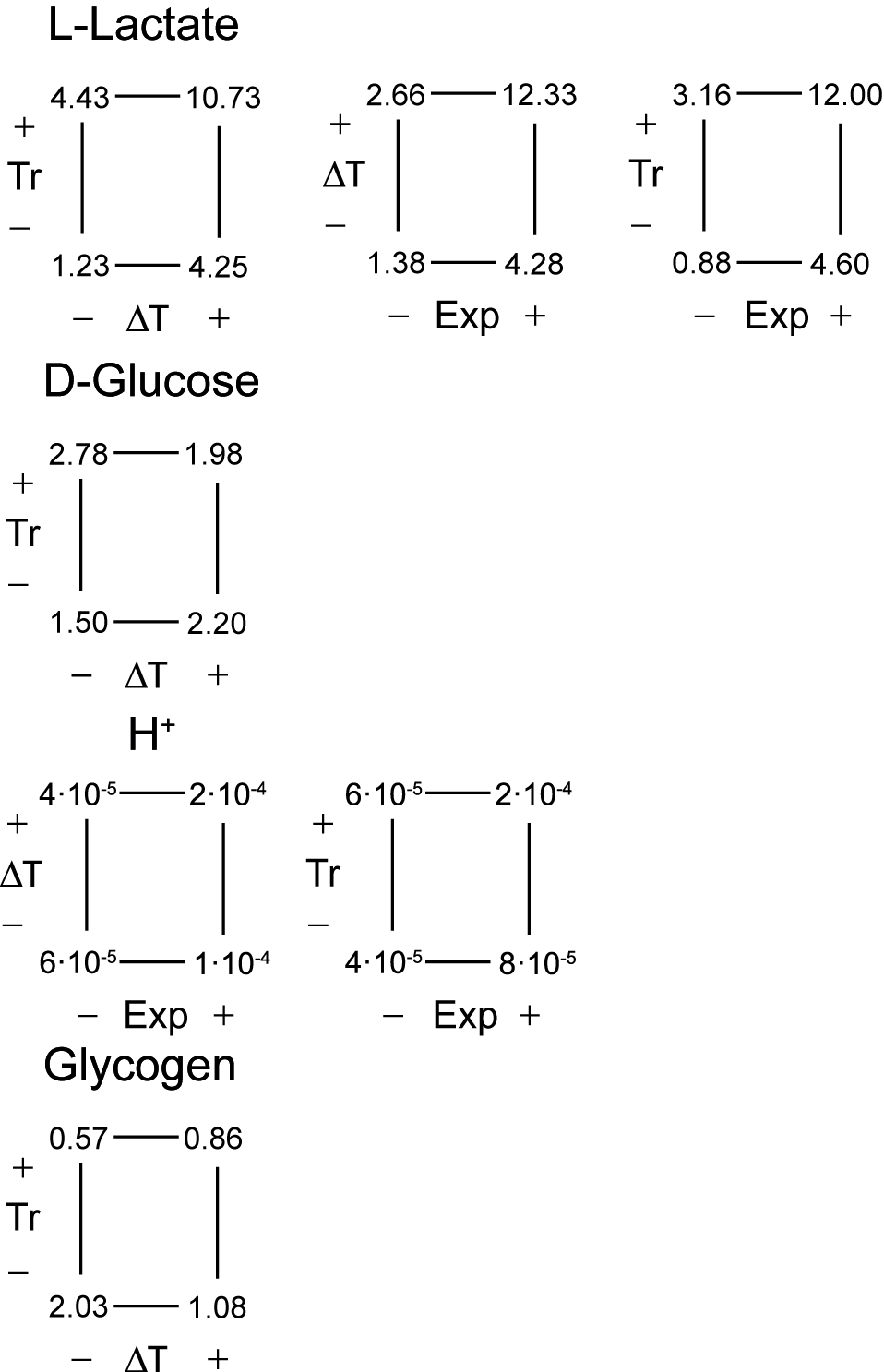

Supplement: Figure S3 — Factorial analysis: numerical values of significant effects of two-factor interactions on Squilla mantis . The factors are: “trawling” (Tr), “thermal shock” (ΔT: Tair - Twater), and “exposure time” (Exp) at two levels (+) and (−), as described in the Materials and Methods section. (TIF) [file pone.0105060.s003.tif]

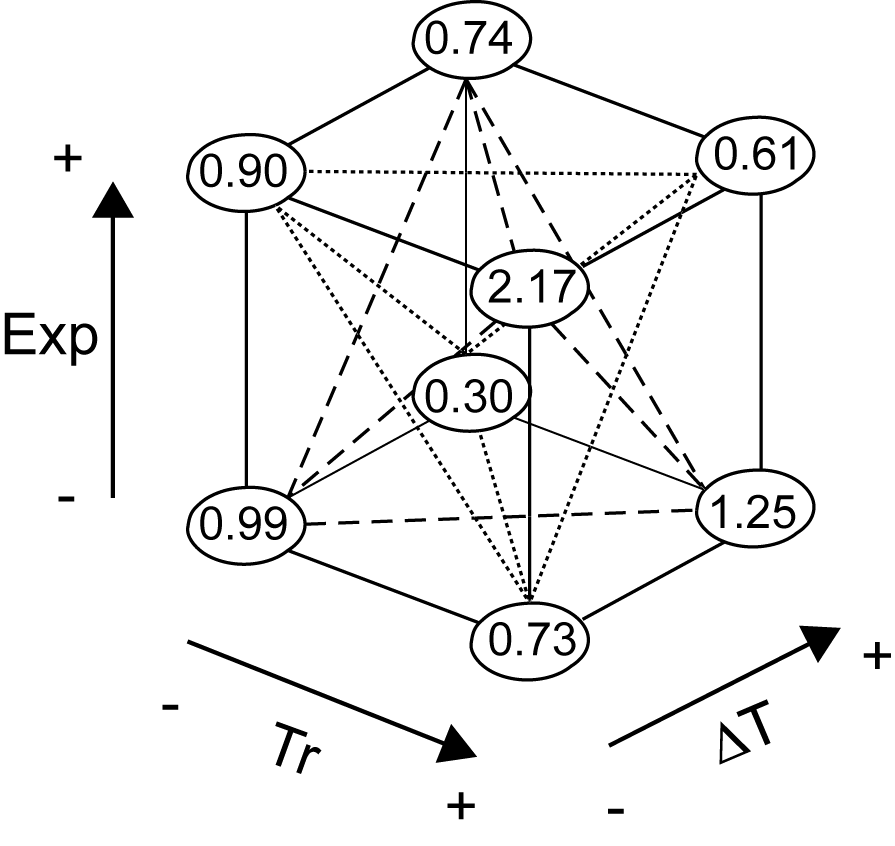

Supplement: Figure S4 — Results of the factorial analysis: numerical values of significant effects of three-factor interactions (ammonia). The factors are: “trawling” (Tr), “thermal shock” (ΔT: Tair - Twater), and “exposure time” (Exp) at two levels (+) and (−), as described in the Materials and Methods section. (TIF) [file pone.0105060.s004.tif]

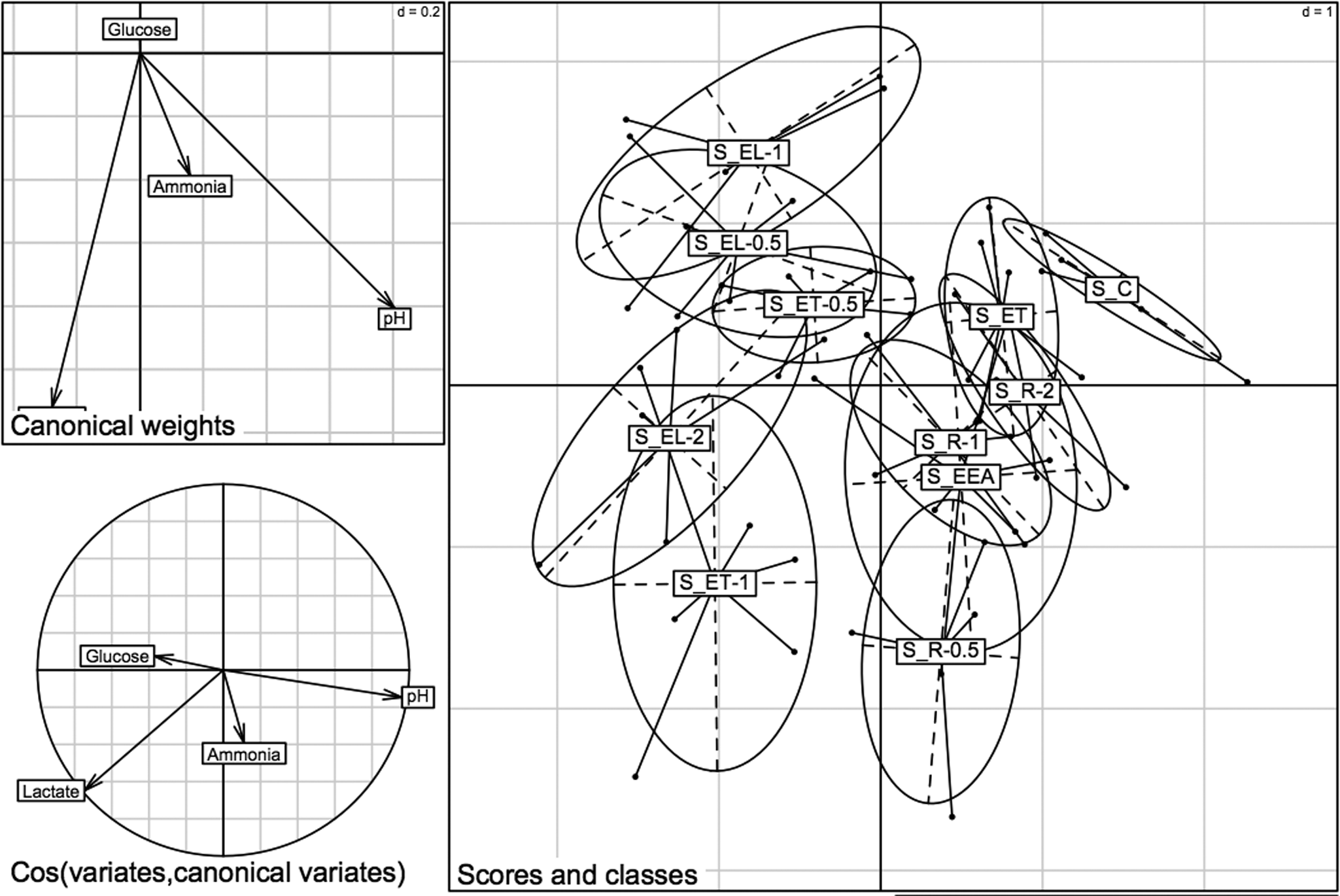

Supplement: Figure S5 — DCA plots applied considering stress indicators in winter and spring experiments. The analysis considered stress-indicator data (glucose, lactate, ammonia, and pH) according to two seasonal datasets (winter: W; spring: S). The figure is a plot composed of i) a plot of the canonical weights (top left), ii) a plot of correlations between discriminant variables and functions (bottom left), and iii) a plot of the canonical scores with ellipses and gravity. C = laboratory control group; EL = emersed groups in lab conditions; ET = trawled groups; EEA = end of exposure to air after trawling in the recovery study; R = individuals exposed to air and re-immersed in tanks with running seawater. 0, 0.5, 1, 2, 4, 8, 12, and 24: time after exposure to air or re-immersion in tanks with running seawater, in hours. (TIF) [file pone.0105060.s005.tif]

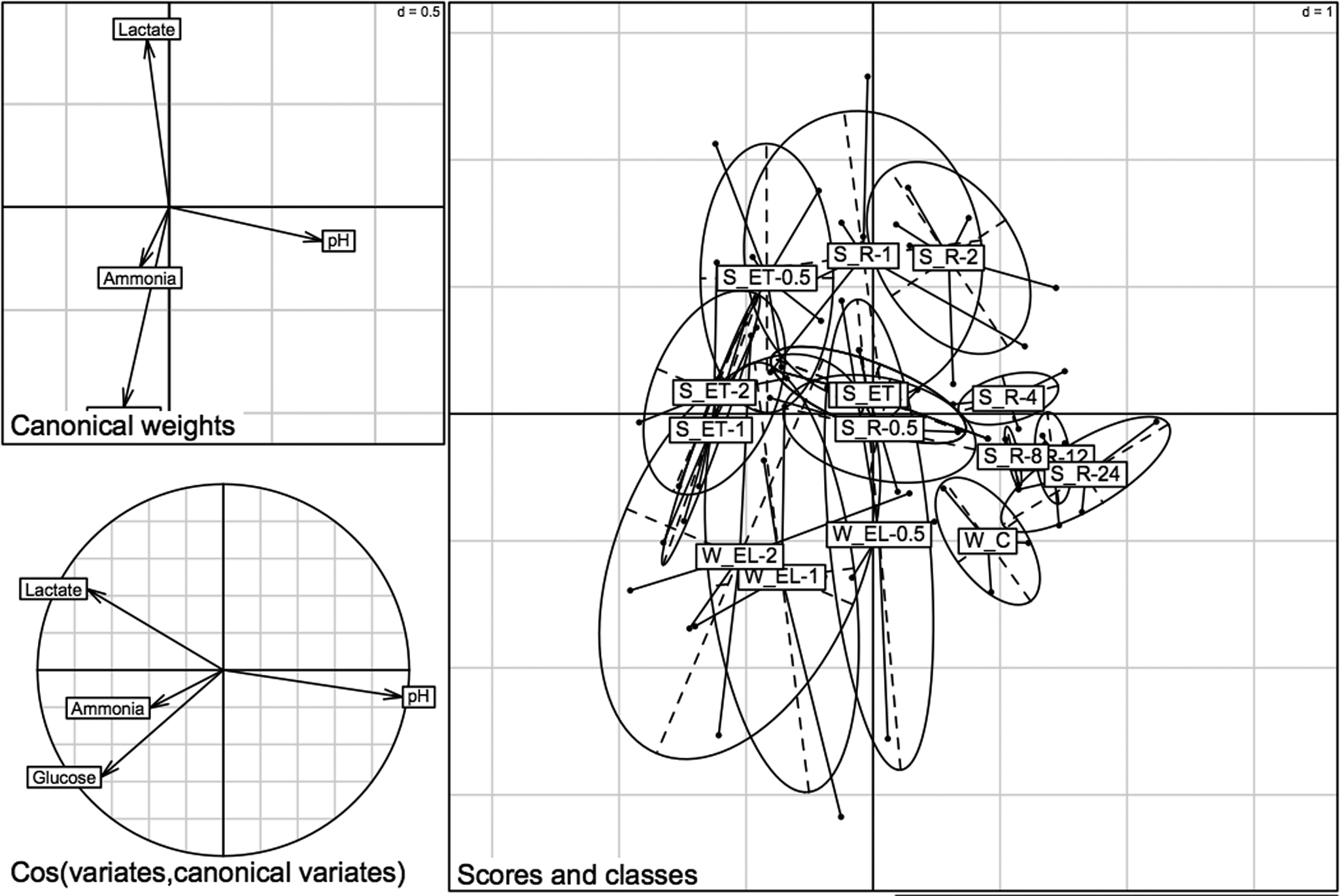

Supplement: Figure S6 — DCA plots applied considering stress indicators in summer experiments. The analysis considered stress-indicator data (glucose, lactate, ammonia, and pH) according to summer seasonal dataset (S). The figure is a plot composed of i) a plot of the canonical weights (top left), ii) a plot of correlations between discriminant variables and functions (bottom left), and iii) a plot of the canonical scores with ellipses and gravity. C = laboratory control group; EL = emersed groups in lab conditions; ET = trawled groups; EEA = end of exposure to air after trawling in the recovery study; R = individuals exposed to air and re-immersed in tanks with running seawater. 0, 0.5, 1, 2: hours after exposure to air or re-immersion in tanks with running seawater, in hours. (TIF) [file pone.0105060.s006.tif]

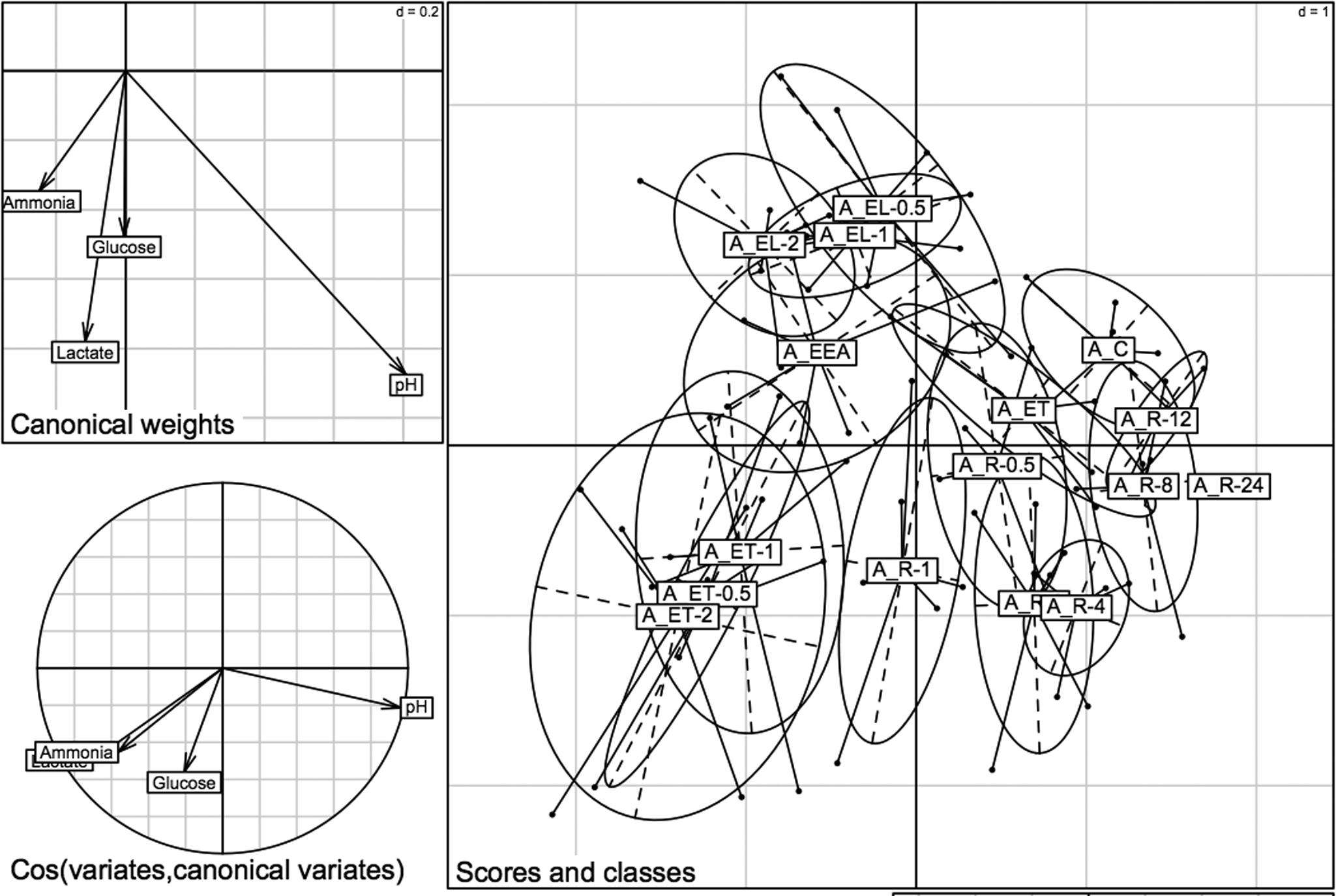

Supplement: Figure S7 — DCA plots applied considering stress indicators in autumn experiments. The analysis considered stress-indicator data (glucose, lactate, ammonia, and pH) according to the autumn seasonal dataset (A).The figure is a plot composed of i) a plot of the canonical weights (top left), ii) a plot of correlations between discriminant variables and functions (bottom left), and iii) a plot of the canonical scores with ellipses and gravity. C = laboratory control group; EL = emersed groups in lab conditions; ET = trawled groups; EEA = end of exposure to air after trawling in the recovery study; R = individuals exposed to air and re-immersed in tanks with running seawater. 0, 0.5, 1, 24, 8, 12, and 24: time after exposure to air or re-immersion in tanks with running seawater, in hours. (TIF) [file pone.0105060.s007.tif]
