# Supplementary material for: Synergistic and Antagonistic Effects of Thermal Shock, Air Exposure, and Fishing Capture on the Physiological Stress of Squilla mantis (Stomatopoda)
Source: PLoS One. 2014 Aug 18;9(8):e105060. doi: 10.1371/journal.pone.0105060 (PMC4136847; doi:10.1371/journal.pone.0105060)
Supplement: Table S1 — Factorial experiment: factors and levels. Summary of experimental conditions (from Table 1) used in the factorial analysis of the effects on physiological parameters of the three factors at two levels. aΔT = Tair-Twater. b -1/+1°C in field and lab experiments, respectively. c +8.0/+10.5°C in lab and field experiments, respectively. (DOC) [file pone.0105060.s008.doc]

**Table S1.** **Factorial experiment: factors and levels.**

|  | Factor | | |
| --- | --- | --- | --- |
| Level | Trawling  (Tr) | Thermal shock  (ΔT^a^) | Time of exposure to air  (Exp) |
| - | Lab experiment | Autumn experiment^b^ | 0 h |
| + | Field experiment | Summer experiment^c^ | 1 h |
