# Supplementary material for: Synergistic and Antagonistic Effects of Thermal Shock, Air Exposure, and Fishing Capture on the Physiological Stress of Squilla mantis (Stomatopoda)
Source: PLoS One. 2014 Aug 18;9(8):e105060. doi: 10.1371/journal.pone.0105060 (PMC4136847; doi:10.1371/journal.pone.0105060)
Supplement: Table S2 — Factorial experiment: design matrix. Summary of the combination of factors and levels for the different 23 experimental conditions used in the factorial analysis. Effects refer to the physiological parameter investigated: L-lactate, D-glucose, the total ammonium nitrogen hemolymph concentration, hemolymph pH, and muscle glycogen concentration. The signs identifying the level of each factor in a given condition are shown in each box. To calculate the effect E, the results Ri for each condition Ci are added, each one with a sign in correspondence of a given column. aExperimental conditions as in Figure 1. bResults of analytical determinations of physiological parameters: concentrations of L-lactate, D-glucose, ammonia nitrogen, H+ (mM), and concentration of glycogen (in mg g−1). c n is the number of the equation described in Text SI (1. Material and Methods) used to calculate the given effect. (DOC) [file pone.0105060.s009.doc]

**Table S2. Factorial experiment: design matrix.**

| **Experimental condition^a^** | **Trawling (Tr)** | **Thermal shock**  **(ΔT)** | **Time of air exposure (Exp)** | **Result^b^** | **Tr*ΔT** | **Tr*Exp** | **ΔT*Exp** | **Tr*ΔT*Exp** |
| --- | --- | --- | --- | --- | --- | --- | --- | --- |
| C_1_ | - | - | - | R_1_ | + | + | + | - |
| C_2_ | + | - | - | R_2_ | - | - | + | + |
| C_3_ | - | + | - | R_3_ | - | + | - | + |
| C_4_ | + | + | - | R_4_ | + | - | - | - |
| C_5_ | - | - | + | R_5_ | + | - | - | + |
| C_6_ | + | - | + | R_6_ | - | + | - | - |
| C_7_ | - | + | + | R_7_ | - | - | + | - |
| C_8_ | + | + | + | R_8_ | + | + | + | + |
| **Effect, *n^c^*** | **E(Tr), *1*** | **E(ΔT)*, 2*** | **E(Exp), *3*** |  | **E(Tr*ΔT), *4*** | **E(Tr*Exp), *5*** | **E(ΔT*Exp), *6*** | **E(Tr*ΔT*Exp), *7*** |
