# Supplementary material for: Synergistic and Antagonistic Effects of Thermal Shock, Air Exposure, and Fishing Capture on the Physiological Stress of Squilla mantis (Stomatopoda)
Source: PLoS One. 2014 Aug 18;9(8):e105060. doi: 10.1371/journal.pone.0105060 (PMC4136847; doi:10.1371/journal.pone.0105060)
Supplement: Table S3 — Experimental values observed in Squilla mantis at different treatment levels. Organisms in aquaria in lab experiment (C); organisms at the end of trawling in field experiments (ET); trawled organisms in field experiments after exposure to air (0.5 hours, EEA); organisms after 2 and 24 hours recovery in water in the field experiments. Values are expressed as a mean ± standard error (n = 6 individuals for each treatment). Significant pairwise comparisons are highlighted between treatments and are shown at the top with bold characters (Mann-Whitney U test, p<0.05). (DOC) [file pone.0105060.s010.doc]

**Table S3. Experimental values observed in *Squilla mantis* at different treatment levels.**

| **Parameter** | **Season** | **Exposure to air data** | | | **Recovery data** | |
| --- | --- | --- | --- | --- | --- | --- |
|  |  | **C** | **ET** | **EEA** | **2h** | **24h** |
| **L-Lactate (mM)** | *Winter* | 1.16 ± 0.69 | - | - | - | - |
|  | *Spring* |  | 7.57 ± 1.39 | 7.57 ± 1.40 | 14.82 ± 1.30 | 0.44 ± 0.24 |
|  | *Summer* | 1.47 ± 0.32 **^EEA, 2h^** | 3.84 ± 0.57 | 7.65 ± 1.31 **^C^** | 4.40 ± 0.38 **^C^** | - |
|  | *Autumn* | 0.29 ± 0.17 **^EEA, 2h^** | 2.47 ± 0.51 | 6.24 ± 0.84 **^C^** | 3.33 ± 0.49 **^C^** | 0.18 ± 0.12 |
| **D-Glucose (mM)** | *Winter* | 1.12 ± 0.10 | - | - | - | - |
|  | *Spring* | - | 0.81 ± 0.15 **^2h^** | 0.81 ± 0.15 **^2h^** | 0.07 ± 0.02 **^ET, EEA^** | 0.41 ± 0.11 |
|  | *Summer* | 1.95 ± 0.13 | 1.82 ± 0.20 | 2.16 ± 0.29 | 1.45 ± 0.26 | - |
|  | *Autumn* | 0.95 ± 0.18 **^EEA^**; **^2h^** | 1.96 ± 0.29 | 1.43 ± 0.24 **^C^** | 3.71 ± 0.33 **^C^** | 2.33 ± 0.35 |
| **Ammonia (mM)** | *Winter* | 1.05 ± 0.61 | - | - | - | - |
|  | *Spring* | - | 1.22 ± 0.24 | 1.22 ± 0.24 | 1.57 ± 0.20 | 1.21 ± 0.10 |
|  | *Summer* | 0.30 ± 0.10 **^EEA^** | 1.25 ± 0.32 | 2.42 ± 0.40 **^C^** | 1.90 ± 0.27 | - |
|  | *Autumn* | 0.99 ± 0.14 **^24h^** | 0.73 ± 0.10 **^24h^** | 0.31 ±0.04 | 0.35 ± 0.02 | 0.08 ± 0.04 **^C, EEA^** |
| **pH** | *Winter* | 7.44 ± 0.07 | - | - | - | - |
|  | *Spring* | - | 7.07 ± 0.06 **^24h^** | 7.07 ± 0.06 **^24h^** | 7.26 ± 0.07 | 7.52 ± 0.05 **^ET, EEA^** |
|  | *Summer* | 7.40 ± 0.07 **^EEA^** | 7.12 ± 0.04 | 7.03 ± 0.05 **^C^** | 7.09 ± 0.09 | - |
|  | *Autumn* | 7.54 ± 0.04 | 7.35 ± 0.09 **^24h^** | 7.13 ± 0.05 **^24h^** | 7.43 ± 0.03 | 7.71 ± 0.02 **^ET, EEA^** |
| **Glycogen (mg ml^-1^)** | *Winter* | - | - | - | - | - |
|  | *Spring* | - | - | - | - | - |
|  | *Summer* | 0.68 ± 0.07 | 0.40 ± 0.02 **^2h^** | 0.44 ± 0.10 | 0.38 ± 0.07 **^ET^** | - |
|  | *Autumn* | 1.45 ± 0.18 | 2.61 ± 0.44 | 2.75 ± 0.59 **^24h^** | 2.13 ± 0.16 | 0.71 ± 0.15 **^EEA^** |
