# Supplementary material for: Synergistic and Antagonistic Effects of Thermal Shock, Air Exposure, and Fishing Capture on the Physiological Stress of Squilla mantis (Stomatopoda)
Source: PLoS One. 2014 Aug 18;9(8):e105060. doi: 10.1371/journal.pone.0105060 (PMC4136847; doi:10.1371/journal.pone.0105060)
Supplement: Table S4 — Exposure to air lab experiment (0–2 hours): 2-Way ANCOVA results. Significant effects are highlighted in bold. (DOC) [file pone.0105060.s011.doc]

**Table S4.** **Exposure to air lab experiment (0-2 hours): 2-Way ANCOVA results.**

| **Parameter** | **Factor** | **df** | **SS** | **MS** | **F** | **p** |
| --- | --- | --- | --- | --- | --- | --- |
| **L-Lactate** | Log (WW) | 1 | 0.15 | 0.15 | 3.1 | 0.09 |
|  | Time | 3 | 3.74 | 1.25 | 26.0 | **< 0.001** |
|  | Season | 2 | 1.41 | 0.71 | 14.7 | **< 0.001** |
|  | Season * Time | 6 | 0.35 | 0.06 | 1.2 | 0.31 |
|  | Error | 50 | 2.40 | 0.05 |  |  |
| **D-Glucose** | Log (WW) | 1 | 0.02 | 0.02 | 1.3 | 0.26 |
|  | Time | 3 | 0.22 | 0.07 | 6.4 | **< 0.001** |
|  | Season | 2 | 0.14 | 0.07 | 5.9 | **< 0.01** |
|  | Season * Time | 6 | 0.10 | 0.02 | 1.4 | 0.22 |
|  | Error | 50 | 0.58 | 0.01 |  |  |
| **Ammonia** | Log (WW) | 1 | 0.01 | 0.01 | 0.3 | 0.57 |
|  | Time | 3 | 0.09 | 0.03 | 1.8 | 0.15 |
|  | Season | 2 | 0.21 | 0.11 | 6.3 | **< 0.01** |
|  | Season * Time | 6 | 0.38 | 0.06 | 3.8 | **< 0.01** |
|  | Error | 50 | 0.84 | 0.02 |  |  |
| **pH** | Log (WW) | 1 | 0.00 | 0.00 | 8.3 | **< 0.01** |
|  | Time | 3 | 0.01 | 0.00 | 51.6 | **< 0.001** |
|  | Season | 2 | 0.00 | 0.00 | 26.3 | **< 0.001** |
|  | Season * Time | 6 | 0.00 | 0.00 | 5.4 | **< 0.001** |
|  | Error | 50 | 0.00 | 0.00 |  |  |
| **Glycogen** | Summer | Kruskal-Wallis Anova test | | | | 0.05 |
|  |  | H ( 3, n = 24) = 7,80 | | | |  |
|  | Autumn | Kruskal-Wallis Anova test | | | | **< 0.05** |
|  |  | H ( 3, n = 23) = 11.04 | | | |  |
