# Supplementary material for: Synergistic and Antagonistic Effects of Thermal Shock, Air Exposure, and Fishing Capture on the Physiological Stress of Squilla mantis (Stomatopoda)
Source: PLoS One. 2014 Aug 18;9(8):e105060. doi: 10.1371/journal.pone.0105060 (PMC4136847; doi:10.1371/journal.pone.0105060)
Supplement: Table S5 — Exposure to air field experiment (0–2 hours): 2-Way ANCOVA results. Significant effects are highlighted in bold. (DOC) [file pone.0105060.s012.doc]

**Table S5. Exposure to air field experiment (0-2 hours): 2-Way ANCOVA results.**

| **Parameter** | **Factor** | **Df** | **SS** | **MS** | **F** | **p** |
| --- | --- | --- | --- | --- | --- | --- |
| **L-Lactate** | Spring | Kruskal-Wallis Anova test | | | | **< 0.01** |
|  | Time | H (3, n = 24) = 14.25 | | | |  |
|  | Summer | Kruskal-Wallis Anova test | | | | 1 |
|  | Season * Time | H (3, n = 18) = 0.00 | | | |  |
|  | Autumn | Kruskal-Wallis Anova test | | | | **< 0.01** |
|  |  | H (3, n = 23) = 11.98 | | | |  |
| **D-Glucose** | Log (WW) | 1 | 0.00 | 0.00 | 0.1 | 0.72 |
|  | Time | 2 | 0.14 | 0.07 | 5.9 | **< 0.01** |
|  | Season | 2 | 0.57 | 0.29 | 23.6 | **< 0.001** |
|  | Season * Time | 4 | 0.09 | 0.02 | 1.9 | 0.13 |
|  | Error | 41 | 0.50 | 0.01 |  |  |
| **Ammonia** | Log (WW) | 1 | 0.01 | 0.01 | 0.8 | 0.38 |
|  | Time | 2 | 0.14 | 0.07 | 7.6 | **< 0.01** |
|  | Season | 2 | 0.16 | 0.08 | 8.5 | **< 0.001** |
|  | Season * Time | 4 | 0.35 | 0.09 | 9.4 | **< 0.001** |
|  | Error | 41 | 0.39 | 0.01 |  |  |
| **pH** | Log (WW) | 1 | 0.00 | 0.00 | 1.8 | 0.19 |
|  | Time | 2 | 0.00 | 0.00 | 37.8 | **< 0.001** |
|  | Season | 2 | 0.00 | 0.00 | 37.8 | **< 0.001** |
|  | Season * Time | 4 | 0.00 | 0.00 | 1.0 | 0.41 |
|  | Error | 41 | 0.00 | 0.00 |  |  |
| **Glycogen** | Log (WW) | 1 | 0.00 | 0.00 | 0.0 | 0.86 |
|  | Time | 2 | 0.01 | 0.01 | 0.3 | 0.73 |
|  | Season | 1 | 0.35 | 0.35 | 17.7 | **< 0.001** |
|  | Season * Time | 2 | 0.18 | 0.09 | 4.5 | **< 0.05** |
|  | Error | 29 | 0.57 | 0.02 |  |  |
