# Supplementary material for: Synergistic and Antagonistic Effects of Thermal Shock, Air Exposure, and Fishing Capture on the Physiological Stress of Squilla mantis (Stomatopoda)
Source: PLoS One. 2014 Aug 18;9(8):e105060. doi: 10.1371/journal.pone.0105060 (PMC4136847; doi:10.1371/journal.pone.0105060)
Supplement: Table S6 — Early phase (0–2 hours) of the recovery process: 2-Way ANCOVA results. Significant effects are highlighted in bold. (DOC) [file pone.0105060.s013.doc]

**Table S6. Early phase (0-2 hours) of the recovery process: 2-Way ANCOVA results.**

| **Parameter** | **Factor** | **Df** | **SS** | **MS** | **F** | **P** |
| --- | --- | --- | --- | --- | --- | --- |
| **D-Glucose** | Log (WW) | 1 | 0.01 | 0.01 | 1.4 | 0.25 |
|  | Time | 3 | 0.01 | 0.00 | 0.4 | 0.77 |
|  | Season | 2 | 1.21 | 0.60 | 73.5 | **< 0.001** |
|  | Season * Time | 6 | 0.54 | 0.09 | 11.1 | **< 0.001** |
|  | Error | 52 | 0.43 | 0.01 |  |  |
| **L-Lactate** | Spring | Kruskal-Wallis Anova test | | | | 0.24 |
|  |  | H (3, n = 24) = 4.23 | | | |  |
|  | Summer | Kruskal-Wallis Anova test | | | | **< 0.05** |
|  |  | H (3, n = 22) = 10.56 | | | |  |
|  | Autumn | Kruskal-Wallis Anova test | | | | 0.17 |
|  |  | H (3, n = 23) = 4.99 | | | |  |
| **Ammonia** | Log (WW) | 1 | 0.00 | 0.00 | 0.1 | 0.74 |
|  | Time | 3 | 0.01 | 0.00 | 0.2 | 0.86 |
|  | Season | 2 | 0.43 | 0.21 | 11.5 | **< 0.001** |
|  | Season * Time | 6 | 0.43 | 0.07 | 3.9 | **< 0.01** |
|  | Error | 52 | 0.96 | 0.02 |  |  |
| **pH** | Log (WW) | 1 | 0.00 | 0.00 | 0.0 | 0.87 |
|  | Time | 3 | 0.00 | 0.00 | 6.2 | **< 0.01** |
|  | Season | 2 | 0.00 | 0.00 | 13.3 | **< 0.001** |
|  | Season * Time | 6 | 0.00 | 0.00 | 1.1 | 0.40 |
|  | Error | 52 | 0.00 | 0.00 |  |  |
| **Glycogen** | Log (WW) | 1 | 0.00 | 0.00 | 0.0 | 0.96 |
|  | Time | 3 | 0.05 | 0.02 | 1.4 | 0.26 |
|  | Season | 1 | 0.71 | 0.71 | 62.8 | **< 0.001** |
|  | Season * Time | 3 | 0.02 | 0.01 | 0.7 | 0.58 |
|  | Error | 37 | 0,42 | 0,01 |  |  |
