# Supplementary material for: Synergistic and Antagonistic Effects of Thermal Shock, Air Exposure, and Fishing Capture on the Physiological Stress of Squilla mantis (Stomatopoda)
Source: PLoS One. 2014 Aug 18;9(8):e105060. doi: 10.1371/journal.pone.0105060 (PMC4136847; doi:10.1371/journal.pone.0105060)
Supplement: Table S7 — Whole recovery experiment: 1-Way ANOVA results. Significant effects are highlighted in bold. (DOC) [file pone.0105060.s014.doc]

**Table S7. Whole recovery experiment: 1-Way ANOVA results.**

| **Parameter** | **Season** | **Factor** | **df** | **SS** | **MS** | **F** | **P** |
| --- | --- | --- | --- | --- | --- | --- | --- |
| **D-Glucose** | Spring |  | Kruskal-Wallis Anova test | | | | **< 0.001** |
|  |  |  | H (7, n = 48) = 29.82 | | | |  |
|  | Autumn | Time | 7 | 0.38 | 0.05 | 5.0 | **< 0.001** |
|  |  | Error | 28 | 0.30 | 0.01 |  |  |
| **L-Lactate** | Spring | Time | 7 | 5.26 | 0.75 | 11.2 | **< 0.001** |
|  |  | Error | 33 | 2.22 | 0.07 |  |  |
|  | Autumn | Time | 7 | 2.44 | 0.35 | 12.6 | **< 0.001** |
|  |  | Error | 28 | 0.78 | 0.03 |  |  |
| **Ammonia** | Spring | Time | 7 | 0.10 | 0.01 | 1.1 | 0.40 |
|  |  | Error | 33 | 0.43 | 0.01 |  |  |
|  | Autumn |  | Kruskal-Wallis Anova test | | | | **< 0.001** |
|  |  |  | H (7, n = 43) = 30.11 | | | |  |
| **pH** | Spring | Time | 7 | 0.00 | 0.00 | 10.4 | **< 0.001** |
|  |  | Error | 33 | 0.00 | 0.00 |  |  |
|  | Autumn | Time | 7 | 0.00 | 0.00 | 21.8 | **< 0.001** |
|  |  | Error | 28 | 0.00 | 0.00 |  |  |
| **Glycogen** | Spring | not measured | | | | | |
|  | Autumn | Time | 7 | 0.32 | 0.05 | 2.2 | 0.07 |
|  |  | Error | 28 | 0.59 | 0.02 |  |  |
