# Supplementary material for: Synergistic and Antagonistic Effects of Thermal Shock, Air Exposure, and Fishing Capture on the Physiological Stress of Squilla mantis (Stomatopoda)
Source: PLoS One. 2014 Aug 18;9(8):e105060. doi: 10.1371/journal.pone.0105060 (PMC4136847; doi:10.1371/journal.pone.0105060)
Supplement: Text S1 — Supporting Information for Materials and Methods. (DOC) [file pone.0105060.s015.doc]

**Synergistic and Antagonistic Effects of Thermal Shock, Air Exposure, and Fishing Capture on the Physiological Stress of *Squilla mantis* (Stomatopoda)**

Saša Raicevich, Fabrizio Minute, Maria Grazia Finoia, Francesca Caranfa, Paolo Di Muro, Lucia Scapolan, Mariano Beltramini

**Supplementary Text SI1. Material and Methods**

**1.1. Analytical determinations**

The hemolymph concentrations of L-lactate and of D-glucose were determined using the enzymatic assays described in [1] and [2], respectively. The following enzymes were used: lactate dehydrogenase (510 U mg^-1^ Sigma Aldrich L2625-12.5KU), hexokinase, and glucose-6P-dehydrogenase (mixed solution from Sigma Aldrich H8629-500UN). For these determinations, the hemolymph was deproteinized according to [3] in order to avoid interference due to hemocyanin. The total ammonium nitrogen concentration was determined colorimetrically using a phenol reagent [4]. The glycogen content in the muscle (in mg g^-1^ fresh weight) was assayed with the anthrone method [5]. In addition, the pH was measured in the total hemolymph with a Radiometer mod. PHM82 microelectrode (Radiometer, Denmark) immediately after sampling, according to the procedure described by other authors [6, 7].

**1.2. Post-emersion recovery**

The pattern of recovery after air exposure was investigated using an experimental design, as in [8]. To this end, a group of n = 48 individuals was sampled after retrieval and exposed to air for 0.5 hours, an interval that is consistent with the duration of the sorting process during commercial fishing activities [9]. At the end of the exposure to air (“EEA”), individuals were introduced into shaded tanks (70 litres) supplied with running seawater kept at sea-bottom temperature. For the spring and winter experiments, the hemolymph was withdrawn from individuals (n = 6) at EEA and at 0.5, 1.0, 2.0, 4.0, 8.0, 12.0, and 24.0 hours after EEA. In the summer experiment, due to the scarcity of live individuals determined by the high post-capture mortality, the hemolymph was sampled at 0.5, 1.0, and 2.0 hours after EEA. In this way, hemolymph was withdrawn from the same number of organisms in all treatments.

**1.3. Effects of salinity shock in controlled conditions**

The effects of salinity on *Squilla mantis* was studied on specimens kept adapted in aquaria at constant temperature (10 °C) and farmed at a salinity of 35 PSU. To simulate a salinity change during a tow-through of different water haloclines, groups of n = 6 individuals were transferred into aquaria at 30, 25, and 20 PSU and kept for 30 minutes before hemolymph withdrawal. A group of n = 6 individuals kept at 35 PSU represented the control group. No mortality was observed within the specimens subjected to such treatment. The linear regression between osmolarity and experimental salinity was estimated. The overall pattern of D-glucose, L-lactate, ammonia, and pH according to different experimental treatments was assessed by means of a 1-way ANCOVA (factor: treatment; covariable: log weight) and post-doc pairwise comparisons were carried out by means of the HSD Tukey test.

**1.4. Factorial Design**

In our factorial analysis, each factor was set at two levels: the (-) and the (+) value. Therefore, our analysis encompasses 2^3^ different conditions resulting from the linear combinations of the two levels of the three factors. As far as “trawling” (Tr) is concerned, the (-) condition includes the dataset from lab experiment since such animals were not caught in the field but acclimatized in aquaria whereas condition (+) refers to specimens caught in the field experiments. For the “thermal shock” (ΔT), the (-) condition includes data of the autumn experiments, where the temperature differences between air and water were -1/+1 °C (in the field and lab experiments, respectively), whereas the (+) condition includes data of the summer experiments, where differences of +8.0/+10.5 °C (in the lab and field experiments, respectively) were recorded. Finally, regarding the “time of exposure to air” (Exp), the (-) value involves organisms not exposed to air (in the lab experiment) or immediately after trawling (in the field experiment), whereas the (+) values refer to organisms exposed to air for 1 hour. The set of conditions tested in the factorial experiment are reported in Table S1. For each factor and level, 6 experimental outputs were available since the same analytical determination was replicated for each of the 6 individuals subjected to a given condition.

It is worth mentioning that the entire dataset includes all possible combinations of levels and variables with analytical results on the same number of organisms (n=6) for the different physiological indicators: L-lactate, D-glucose, ammonium nitrogen, H^+^ in hemolymph, and muscle glycogen.

The effects of single factors (Tr, ΔT, Exp) and the “main effect” of the factor, E(x), were obtained by algebraically averaging all results R_i_, each one relative to each i^th^ experimental condition, as summarized in the design matrix shown Table S2 [10].

Thus, the following values were calculated according to Table S2:

E(Tr) =
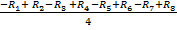
 (1)

E(ΔT) =
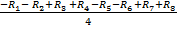
 (2)

E(Exp) =
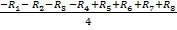
 (3)

The second- and third-order effects due to the interaction between factors were obtained by the same procedure:

E(Tr **.** ΔT) =
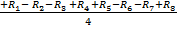
 (4)

E(Tr **.** Exp) =
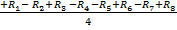
 (5)

E(ΔT **.** Exp) =
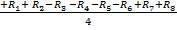
 (6)

E(Tr **.** ΔT **.** Exp) =
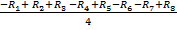
 (7)

It is worth noting that in calculating the main effects of each single factor with Equations 1-3, each numerical values of R_i_ is represented with a sign corresponding to the level that the factor assumes in a given condition C_i_. In calculating the interactions between factors with Equations 4-7, the signs of R_i_ result from the multiplication of the signs of the interacting factors in each condition C_i_.

The variance relative to each effect was calculated as described by [10]:

V_effect_ =
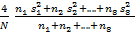
, (8)

where N = 48 (6 individuals, 8 conditions) and n_1…8_ and s^2^_1…8_ are the degrees of freedom and the variance of the i^th^ experimental condition. The estimated standard error is calculated as
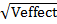
. The significance of the effects was evaluated by comparing E(i) values with their estimated standard error according to the Student’s *t-*test for 8 degrees of freedom.

***Multivariate analysis.***

The significance of the discriminant functions was assessed by applying a Monte Carlo test on the DCA analysis (i.e., the test of the sum of the discriminant analysis eigenvalues, divided by the rank; the non-parametric version of the Pillai Test) by means of 999 permutations [11]. All the statistical analysis were carried out by means of the R software [12] and the software package ade4 [13-15].

**References**

1. Gutmann I, Wahlefeld AW (1974) L-(+)-lactate: determination with lactate dehydrogenase and NAD+. In: Bergmeier HU editor. Methods of Enzymatic Analysis. Academic Press, New York.
2. Kunst A, Draeger B, Ziegenhorn J (1983) UV-methods with hexokinase and glucose-6-phosphate dehydrogenase. In: Bergmeyer HU editor. Methods of Enzymatic Analysis. Verlag Chemie, Weinheim.
3. Ridgway ID, Taylor AC, Atkinson RJA, Chang ES, Neil DM (2006) Impact of capture method and trawl duration on the health status of the Norway lobster, *Nephrops norvegicus*. J Exp Mar Biol Ecol 339:135–147.
4. Solorzano L (1969) Determination of ammonia in natural waters by phenolhypochlorite method. Limn Ocean 4:799-801.
5. Carroll NV, Longley RW, Roe JH (1956) The determination of glycogen in liver and muscle by use of anthrone reagent. J Biol Chem 220: 583-593.
6. Bergmann M, Taylor AC, Moore PG (2001) Physiological stress in decapods crustaceans (*Munida rugosa* and *Liocarcinus depurator*) discarded in the Clyde *Nephrops* fishery. J Exp Mar Biol Ecol 259: 215-229.
7. Qadri SA, Camacho J, Wang H, Taylor JR, Grosell M, Worden MK (2007) Temperature and acid-base balance in the American lobster *Homarus americanus*. J Exp Biol 210:1245-1254.
8. Raicevich S, Giomi F, Pranovi F, Giovanardi O, Di Muro P, Beltramini M (2011) Onset of and recovery from physiological stress in *Liocarcinus depurator* after trawling and air exposure under different seasonal conditions. Hydrobiologia 664: 107-118.
9. Pranovi F, Raicevich S, Franceschini G., Torricelli P Giovanardi O (2001) Discard composition and damage to non-target species in the “rapido” trawl fishery. Marine Biology 139:863-875.
10. Box GEP, Hunter WG., Hunter JS. (1978) Statistics for experimenters, Wiley, New York.
11. Manly BFJ (1991) Randomization and Monte Carlo methods in biology. Chapman and Hall, London.
12. R Core Team (2012) R: A language and environment for  statistical computing.  R Foundation for Statistical Computing, Vienna, Austria. ISBN 3-900051-07-0, URL [http://www.R-project.org/.)](http://www.r-project.org/.))
13. Chessel D, Dufour A B, Thioulouse J (2004) The ade4 package-I-One-table methods. R News 4: 5-10**.**
14. Dray S, Dufour AB (2007) The ade4 package:  implementing the duality diagram for ecologists. J Stat Soft 22(4): 1-20.
15. Dray S, Dufour AB, Chessel D. (2007) The ade4 package-II: Two-table and K-table methods. R News. 7(2): 47-52).
