# Supplementary material for: Synergistic and Antagonistic Effects of Thermal Shock, Air Exposure, and Fishing Capture on the Physiological Stress of Squilla mantis (Stomatopoda)
Source: PLoS One. 2014 Aug 18;9(8):e105060. doi: 10.1371/journal.pone.0105060 (PMC4136847; doi:10.1371/journal.pone.0105060)
Supplement: Text S2 — Supporting Information for Results. (DOC) [file pone.0105060.s016.doc]

**Synergistic and Antagonistic Effects of Thermal Shock, Air Exposure, and Fishing Capture on the Physiological Stress of *Squilla mantis* (Stomatopoda)**

Saša Raicevich, Fabrizio Minute, Maria Grazia Finoia, Francesca Caranfa, Paolo Di Muro, Lucia Scapolan, Mariano Beltramini

**Supplementary Text SI2. Results**

**2.1. Reversibility of physiological stress: post-emersion recovery in field experiments.**

In order to assess the reversibility of physiological stress induced by trawling and emersion, specimens at the end of exposure to air were reimmersed in water, as described in the Materials and Methods section. As a general result, the trends versus time of all parameters clearly show the recovery of the physiological parameters toward values closer to those observed at the beginning of the exposure to air or the lab conditions, pointing to a negligible effect of the onboard storage on sampled individuals. In addition to the time courses shown in Figure S1, the experimental values observed with organisms from aquaria (C), at the end of trawling (ET) at the end of the time of exposure to air (0.5 hours; EEA), and 2 and 24 hours after recovery in water are reported in Table S3, along with the results of pairwise statistical comparisons. These results help one to better visualize the effects of trawling, emersion, and recovery on the studied parameters. Tables S6 and S7 summarize the results of statistical analysis relative to the early phases of recovery (0-24 hours) and relative to the whole recovery experiment.

In the early phases of the recovery process (0-2 hours after reimmersion in water), the L-lactate showed significantly different values over time only in the summer experiment (Kruskal-Wallis non-parametric ANOVA, p < 0.05) with a decreasing trend. However, in this season, both EEA and the 2 hour treatments had significantly higher values than the control group (Mann-Whitney U test, p < 0.01 and p < 0.05, respectively), showing that the imbalance induced by trawling and exposure to air was not yet fully recovered in this parameter in such a short reimmersion time. A similar pattern in L-lactate values in the comparison among the C and EEA and 2 hour groups was observed in the autumn experiments (Mann-Whitney U test, C vs. EEA: p < 0.001; C vs. 2 hour: p < 0.05), although the concentration of this metabolite was slightly lower as compared with the summer treatments. During spring, an increase in this parameter was observed, 1 hour after the beginning of the recovery experiment, but no significant trend over time was recorded, probably due to the high variability in the data.

When considering the D-glucose concentration, different trends over time in the early recovery phases have been observed (2-way ANCOVA, season · time: p < 0.001). Indeed, in spring and summer, a tendency toward a D-glucose decrease has been recorded (although only in spring at 2 hour, significantly lower values than those recorded at EEA and ET were observed; Mann-Whitney U test, p < 0.05 in both the comparisons). On the other hand, an increase in D-glucose was recorded in autumn, with values significantly higher than the C group at the beginning (EEA) and 2 hours after the beginning of the recovery experiment (Mann-Whitney U test, C vs. EEA: p < 0.01; C vs. 2 hours: p < 0.05). Over the 24 hours, both in the spring and the autumn. the variation of D-glucose was significant (Spring, Kruskal-Wallis ANOVA, time: p < 0.001; autumn, 1-way ANOVA, time: p < 0.001) and values at the end of the recovery did not differ significantly from the C values. As far as the pH is concerned, both considering the early recovery phase (0-2 hours) and the full recovery (0-24 hours), a tendency toward the re-establishment of unstressed conditions in all considered seasonal experiments has been detected. These trends were similar in the early phases of the recovery process (2-way ANCOVA, time: p <0.01; season: p < 0.001), although the autumn values showed a less marked acidosis compared with the other seasonal experiments. In the full seasonal recovery experiments (0-24 hours, spring, and autumn), changes in this parameter were recorded over time (1-way ANOVA, time: p < 0.001 in both seasons). At the end of the recovery experiment (24 hours), the pH values were significantly higher than those at the ET and EEA both in the spring and the autumn (Mann-Whitney U test, spring: 24 hours vs. ET and 24 hours vs. EEA, p < 0.001; autumn: 24 hours vs. ET, p < 0.01; 24 hours vs. EEA, p < 0.001), with average values of about 7.5-7.7 compared with those recorded at EEA (about 7.10), showing that this parameter returned to its unstressed value at the end of the experiments.

As in the case of the field exposure to air experiments, ammonia showed different temporal trends in the early recovery phases that differed according to the season (2-way ANCOVA, season · time: p < 0.01). The highest values were recorded in summer, when a significantly higher ammonia concentration was observed at EEA as compared with C (1.9 mM vs. 0.30 mM; Mann-Whitney U test, p < 0.01). While in spring the overall recovery experiment did not show a significant temporal trend, in autumn a significant temporal pattern was observed (Kruskal-Wallis non-parametric ANOVA, p < 0.001), with very low values (0.08 mM) that were significantly lower than the C and ET values (Mann-Whitney U test, 24 hours vs. C: p < 0.001; 24 hours vs. ET: p < 0.01)

The only physiological parameter that did not show any significant temporal trend in the recovery experiments was glycogen. In this case, a different seasonal pattern consistent with the exposure to air field experiment was, however, detected. In detail, glycogen showed higher concentrations in autumn compared with summer (2-way ANCOVA, season: p < 0.001). It is worth noting that at the end of the autumn recovery experiment, glycogen showed significantly lower values that at the EEA, thus indicating that this source of energy was depleted in this season after the recovery (Mann-Whitney U test, p < 0.05). At the same time, in summer, significant lower values in glycogen were observed at the end of the recovery process as compared with the ET values (Mann-Whitney U test, 2 hours vs. ET, p < 0.05).

**2.2. Effects of salinity shock in controlled conditions**

The results reported in Figure S2 indicate an osmoconformer response of *Squilla mantis* to salinity changes. Upon decreasing the environmental salinity, the internal osmotic pressure decreased linearly, within the investigated range (Osmolarity = 0.3073 + 0.0199 * Salinity; R^2^ = 0.56, p < 0.001). All investigated parameters varied significantly over different salinity ranges (1-way ANCOVA, L-Lactate and pH: p < 0.001; D-glucose and ammonia: p < 0.05). D-glucose increased in all the three low-salinity treatments, reaching significantly higher values in all treatments as compared with the controls (HSD Tukey’s test, p < 0.05 in all comparisons) . On the other hand, the pH value recorded at the lowest experimental salinity was significantly lower than all the others experimental treatments (HSD Tukey’s test, 20 PSU vs. 25 PSU and control, p <0.05; 20 PSU vs. 30 PSU, p < 0.001). While ammonia showed a tendency to increase with decreasing salinity with a significantly lower concentration in the comparison between 20 PSU and the experimental control (HSD Tukey’s test, p < 0.05), L-lactate fluctuated and reached very high values at 20 and 30 PSU, significantly higher then those recorded for the control and 25 PSU groups (HSD Tukey’s test, 20 PSU vs. 25 PSU and control, p < 0.05; 30 PSU vs. 25 PSU and control, p < 0.001).

**2.3 Multivariate analysis.**

DCA plots applied considering stress indicators in winter and spring, summer, autumn experiments are reported in Supplementary Figures S5-S7. Overall, a pattern consistent with univariate analysis was observed. In the lab, control groups were characterized by high pH values, while further exposure to air in such conditions led to the increase in glucose and acidosis. However, the displacement from control individuals differed between emersed and trawled and emersed groups, as highlighted by the different position of the centroids of treatment groups in the PCA. Indeed, individuals collected after trawling and then exposed to air showed a displacement from homeostasis conditions, but in a different direction compared with lab-emersed stomatopods, showing a further decrease in pH and an increase in lactate and, in both summer and autumn, glucose and ammonia.

Recovery from trawling and exposure to air followed a pattern with an increase in lactate and glucose in the first part of the recovery process that was later characterized by a decrease in such parameters and an increase in pH, with nearly a re-establishment of physiological conditions very close to those of the control groups.
